# Efficient and Selective Electrochemical CO_2_ to Formic Acid Conversion: A First-Principles Study of Single-Atom and Dual-Atom Catalysts on Tin Disulfide Monolayers

**DOI:** 10.1021/acs.jpcc.4c02283

**Published:** 2024-09-17

**Authors:** Guanming Chen, Margherita Buraschi, Rashid Al-Heidous, Satyanarayana Bonakala, Fedwa El-Mellouhi, Clotilde S. Cucinotta

**Affiliations:** † Department of Chemistry, and Thomas Young Centre, Imperial College London, White City Campus, London W12 0BZ, U.K.; ‡ Qatar Environment and Energy Research Institute, Hamad Bin Khalifa University, PoBox 34110, Doha, 2662, Qatar

## Abstract

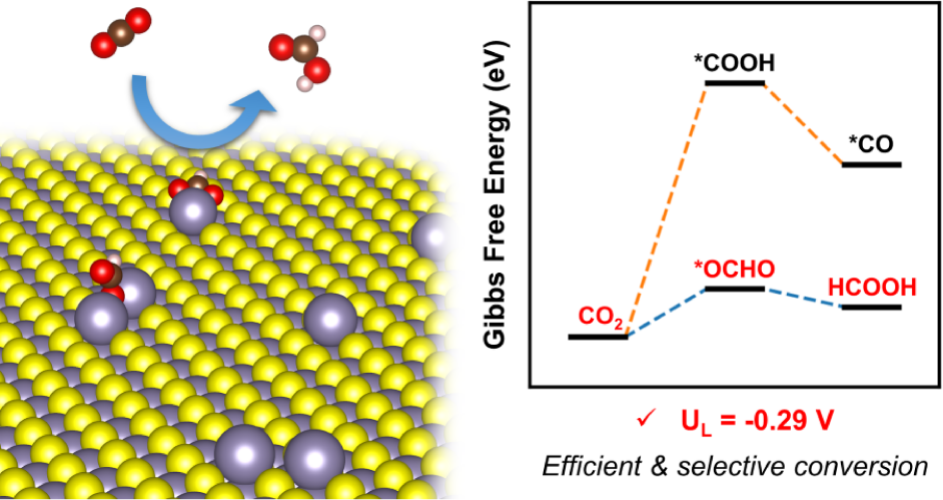

Electrochemical CO_2_ reduction reaction (CO_2_RR) is a sustainable approach to recycle CO_2_ and address climate issues but needs selective catalysts that operate at low electrode potentials. Single-atom catalysts (SACs) and dual-atom catalysts (DACs) have become increasingly popular due to their versatility, unique properties, and outstanding performances in electrocatalytic reactions. In this study, we used Density Functional Theory along with the computational hydrogen electrode methodology to study the stability and activity of SACs and DACs by adsorbing metal atoms onto SnS_2_ monolayers. With a focus on optimizing the selective conversion of CO_2_ to formic acid, our analysis of the thermodynamics of CO_2_RR reveals that the Sn-SAC catalyst can efficiently and selectively catalyze formic acid production, being characterized by the low theoretical limiting potentials of −0.29 V. The investigation of the catalysts stability suggests that structures with low metal coverage and isolated metal centers can be synthesized. Bader analysis of charge redistribution during CO_2_RR demonstrates that the SnS_2_ substrate primarily provides the electronic charges for the reduction of CO_2_, highlighting the substrate’s essential role in the catalysis, which is also confirmed by further electronic structure calculations.

## Introduction

Since human society has extensively used fossil fuels , atmospheric CO_2_ levels have been rising significantly. This increase in CO_2_ contributes to the greenhouse effect, leading to climate change and various natural disasters. Consequently, it is crucial to find a balance between CO_2_ emissions and their elimination. A promising strategy to reduce the concentration of CO_2_ in the atmosphere is to recycle CO_2_ into valuable feedstock for the chemical industry.^1^ Traditional methods, such as the reverse water–gas shift reaction and the Fischer–Tropsch process, have been used in industries for the large-scale conversion of CO_2_ into hydrocarbons and methanol. However, these methods often require high temperatures and pressures, making them energy-intensive and not aligned with net-zero carbon emission goals. Therefore, finding an alternative way for recycling CO_2_ under mild conditions is essential. Electrochemical CO_2_ reduction reaction (CO_2_RR) is gaining interest because it can operate at room temperature and utilize renewable electricity from sources like wind or solar energy.

The electrochemical CO_2_RR on metal electrodes was first investigated quantitatively by Hori et al. in 1985.^2^ He later classified metal electrodes based on their selectivity toward different CO_2_RR products, and it emerged that Cu is the only metal that can produce valuable hydrocarbons and alcohols as the main products.^3^ However, a significant issue with pure metal electrodes for CO_2_RR is poor selectivity toward desirable products. This is due to the complex nature of CO_2_RR, which involves multiple intermediates and products.^4,5^ In addition, the high overpotential of Cu electrode for electrochemical CO_2_RR (1.2 V for methane) limits its application.^1^ Hence, optimizing the catalyst structure and designing new catalysts for better applications in CO_2_RR are essential.

The optimization of catalysts can be achieved by increasing the utilization rate of metal atoms and enhancing the intrinsic activity of the catalytic centers.^4,6^ To achieve this, many innovative catalysts, such as nanostructured materials and bimetallic alloys, have been developed.^1^ Among these systems, single-atom catalysts (SACs) combine the advantages of both strategies. In an SAC, metal atoms are ideally well dispersed on supporting substrates to form catalytic centers at the single-atom level. Single metal centers are usually coordinated by heteroatoms, such as N, O, and S, which stabilize the metal centers on the substrate and tune the electronic properties of the active sites.^4,7^ The unique structure of SACs leads to a maximized atomic utilization rate, fine-tuned electronic properties, and good catalytic performance.^4^ Recent research has reported that SACs exhibit high performance in electrochemical CO_2_RR. The substrates of reported SACs are diverse, and include metal–organic frameworks,^8−10^ two-dimensional (2D) materials,^11,12^ and large organic heterocyclic molecules such as phthalocyanine.^13−15^ Transition metals (TMs), such as Fe, Ni, and Cu, were employed as the catalytic centers in these SACs. These catalysts can be used to cover a wide range of CO_2_RR products and displayed good selectivity. However, SACs also have limitations. Aggregation can occur easily during their synthesis,^6^ so there is a need for adequate preparation methods. Additionally, SACs respect the linear scaling relations (LSRs) between the adsorption energies of different reaction intermediates, which poses potential issues.^16^ Indeed, due to these LSRs, it is not possible to adjust the binding energy of one particular reaction intermediate without affecting the others. Therefore, it is difficult to optimize the selectivity of CO_2_RR by trying to manipulate the binding strength of different intermediates on the catalytic surfaces.

Recently, the concept of dual-atom catalysts (DACs) was introduced,^6^ inspired by natural metalloenzymes containing two metal atom centers, which are capable of reducing molecules, such as CO_2_ and N_2_.^17,18^ A DAC is a catalyst with two adjacent metal atoms acting as the catalytic centers. Compared to SACs, DACs possess similar advantages but with more flexibility in structure and a wider range of combinations for the dual metal centers.^6^ These dual-atom configurations can produce unique electronic properties and break LSRs, which resulted in enhanced performances in electrochemical CO_2_RR.^19^ DACs with Fe–Co, Fe–Ni, and Co–Zn sites have demonstrated more efficient CO_2_ conversions than SACs containing just one of these metals.^20−22^ In addition, the dual-atom site has the potential to facilitate the dimerization of reaction intermediates to give multicarbon molecules as final products.^23^

However, DACs are typically less stable than SACs due to the lower coordination of the dual-metal sites, which results in challenges for preparation in experiments and a shorter catalyst lifetime.^6^ Also, there is a lack of understanding of the unique properties of dual-atom sites and their effects on catalysis. Addressing the instability issue and further investigating the mechanisms behind DACs catalytic activity are essential for their rational design.

In electrochemical CO_2_RR, hydrocarbons and alcohols are highly sought-after products. However, formic acid, despite being an essential feedstock in the chemical industry, has received less attention. This study aims to investigate formic acid production through electrochemical CO_2_RR, by exploiting the unique properties of the SnS_2_ substrate.

Notably, Sn is one of the few nontoxic metals able to predominantly produce formic acid in CO_2_RR. The dichalcogenide derivative, tin disulfide (SnS_2_), is a 2D material consisting of stacked monolayers held together through van der Waals interactions. The large surface area of SnS_2_ makes it a good substrate for supporting atomic catalytic centers. Experimental work has shown that when exfoliated into monolayers, SnS_2_ selectively aids the electrochemical CO_2_RR production of formic acid.^24^ In addition, it is possible to modify SnS_2_ monolayers by doping with other metal atoms.^25,26^ In theoretical work, models have been proposed in which different metal atoms are adsorbed on the surface of SnS_2_ monolayer,^27−29^ offering a variety of catalytic scenarios.

In this study, we selected an SnS_2_ monolayer as the substrate to develop SACs and DACs. We constructed several models for these catalysts adsorbing different metal atoms, namely Ag, Cu, Sn, and Zn, over the basal sulfur plane of an SnS_2_ monolayer and simulated their catalytic performances in CO_2_RR under electrochemical conditions. The selection of metals included those that are nontoxic, abundant on Earth, and selectively give different CO_2_RR products based on experimental work:^3^ Sn for formic acid, Ag and Zn for CO, and Cu for alcohols and hydrocarbons. We investigated the CO_2_RR performance of these metals when adsorbed on an SnS_2_ monolayer as atomic-level sites. We also investigated the stability of these systems to assess their feasibility for experimental synthesis.

It is worth noting that there are two common polytypes of SnS_2_, 2H- and 4H-, which differ in the stacking of the SnS_2_ monolayers (see Figure S1). In this study, we used the 2H-SnS_2_ structure as our reference for bulk SnS_2_.

## Computational Details

Density functional theory (DFT) calculations were performed, as implemented in the Quickstep module of CP2K code,^30^ which uses a mixed Gaussian and plane waves (GPW)^31^ basis set, and Goedeker-Teter-Hutter (GTH) pseudopotentials to describe the core electrons.^32−34^ The molecular optimized (MOLOPT) basis sets,^35^ and a CUTOFF energy of 450 Ry for the plane-wave basis sets were used. A 9 × 9 × 5 Monkhorst–Pack grid of k-points^36^ was used to sample the first Brillouin zone of bulk SnS_2_. With this selection of the simulation parameters the error in total energy was less than 1 × 10^–4^ eV per atom. To simulate an isolated 2D SnS_2_ monolayer, a 20 Å vacuum gap was inserted along the *z* direction in the optimized bulk SnS_2_ cell. The Perdew–Burke–Ernzerhof (PBE) exchange-correlation functional^37^ was adopted, with Grimme’s D3 dispersion corrections (with Becke-Johnson damping),^38^ which are needed to describe van der Waals interactions between SnS_2_ monolayers. The atomic positions were fully relaxed until the geometry and forces changed by less than 1.6 × 10^–3^ Å and 2.3 × 10^–2^ eV/Å, respectively. We observed that the PBE-D3 functional can reproduce the 2D structure of an SnS_2_ monolayer but does not perform well in describing interlayer distance in bulk SnS_2_ and the band gaps of SnS_2_-related systems. Therefore, the Heyd-Scuseria-Ernzerhof (HSE) hybrid functional^39^ was used for calculations of electronic structures because it provides more accurate results for these computed quantities (see Tables S1 and 2). To describe band gaps more accurately, we used 0.08 for the screening parameter of the range-separated Coulomb potential (ω), instead of 0.11 used in the classic HSE06 functional^40^ (see Table S3). We noticed that the functional used for geometry optimization has little effect on the structure and band gap of an SnS_2_ monolayer. Therefore, considering that PBE is computationally much lighter than HSE, we performed geometry optimizations at the PBE-D3 level and evaluated the energetics and electronic structures at the HSE-D3 level based on PBE-D3 optimized geometries.

The Gibbs free energy (*G*) was calculated using the following equation:
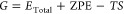
1where *E*_Total_ is the DFT energy, ZPE is the zero-point energy, *T* is temperature, and *S* is the entropy of the system. ZPE and entropies were calculated using the harmonic approximation from the vibrational frequencies computed with the CP2K code. Notably, the results were not affected by the inclusion of low frequencies. Indeed, excluding the lowest three frequencies in the calculations of ZPE and entropy calculations resulted in revised adsorption free energies and associated limiting potentials differing by less than 0.04 eV.

The adsorption free energy of each *C_*x*_H_*y*_O_*z*_ intermediate along the CO_2_RR, Δ*G*_ads_, was evaluated relative to the clean catalyst surface and gas-phase H_2_, H_2_O, and CO_2_. This avoids the need to simulate O_2_, whose ground state is a triplet, which is poorly described by DFT methods. More specifically, the following equation was used:
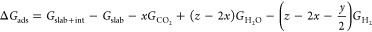
2where *G*_slab+int_ and *G*_slab_ are the free energies of the slab with the adsorbed intermediate and the clean slab, respectively.

The free energy change of each step in the CO_2_RR pathways was calculated as the change in Δ*G*_ads_ between the initial and final states of each elementary step. To simulate the effect of an applied potential and pH under electrochemical conditions, the computational hydrogen electrode (CHE) approach proposed by Nørskov^41^ was adopted. The free energy at the applied electrode potential *U* can be simply adjusted by shifting the free energy by *neU*. Therefore, the free energy at potential *U* with the pH effect of the aqueous reaction environment can be calculated as:
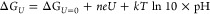
3where *n* is the number of electrons transferred in the electrochemical reaction, *e* is the elementary charge, and *k* is the Boltzmann constant. Variations in pH were used to calculate the Pourbaix diagrams, and for thermodynamic analysis of the electrochemical reaction profiles, the free energies were calculated under pH = 0. The method proposed by Chan and Nørskov^42^ was used to estimate the effects of changing electrode potentials on the reaction energy, and the corrections were small enough to be negligible (see Table S5). The effect of liquid water is accounted for by directly adding ad-hoc corrections to the adsorption free energies.^43,44^ Specifically, for SACs and DACs, the adsorption of *COOH and *CO is stabilized by 0.1 eV in the liquid phase, while the adsorption of *OCHO and *H may not be affected.^45^ Moreover, the configuration when the surface is covered by *OH or *H_2_O at the operating potential is approximately stabilized by 0.2 eV. These corrections were directly applied to our calculated adsorption free energies.

The limiting potential (*U*_*L*_) is the smallest theoretical electrode potential required to have all electrochemical reaction steps decreasing in free energy, which is determined by the largest free energy change among all electrochemical steps at zero electrode potential:^45^
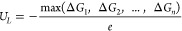
4The reaction pathway with the lowest *U*_*L*_ was regarded as the most favored one among all CO_2_RR pathways, and the selectivity of CO_2_RR on the catalysts could be evaluated. It is worth noting that our evaluation of CO_2_RR selectivity based on thermodynamics offers qualitative results of potentially feasible pathways and products. To determine the actual rates of production, further quantitative analysis, including the evaluation of activation barriers between possible intermediates, would be required. This calculation is out of the scope of this paper, which focuses on determining the possible open pathways toward formic acid evolution.

## Results and Discussion

### SAC and DAC Models

**Figure 1:**
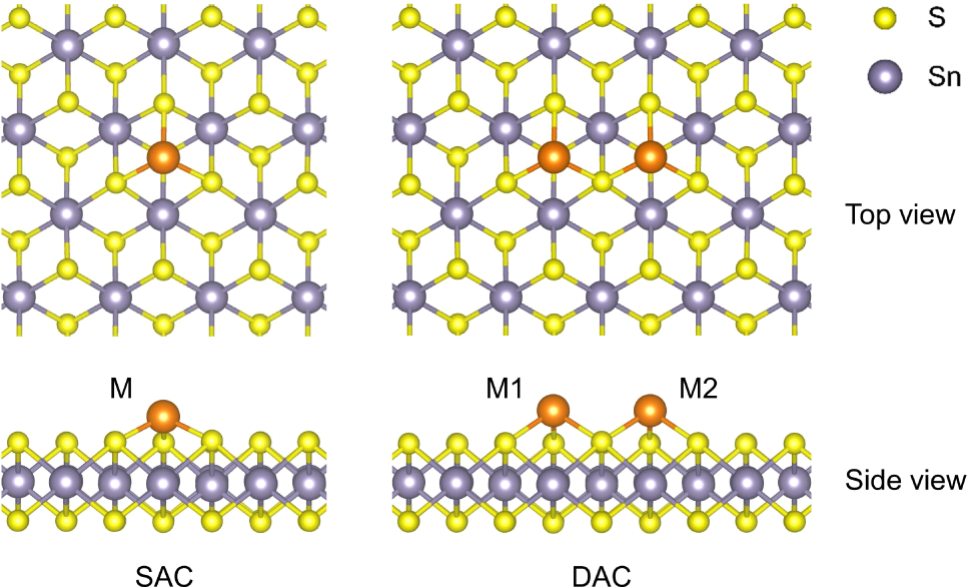
Top and side views of the structures of the developed adsorption models around the active centres on the SnS_2_ monolayer. M, M1, and M2 label the adsorbed metal atoms. In all our models M1 = M2. Blue-gray, yellow, and orange balls represent Sn, S and metal atoms, respectively.

The adsorption models we constructed for SACs and DACs are shown in Figure 1. To manage the complexity of computational screening and ensure the feasibility of experimental preparation, we focused only on homonuclear DACs. We named the SnS_2_ substrate decorated with one isolated metal atom, M, as M-SAC, and the one with two atoms as M-DAC, where M = Ag, Cu, Sn, and Zn. Each metal atom in these models is coordinated with three sulfur atoms, which is the most stable adsorption configuration on the surface of an SnS_2_ monolayer.^46^ To construct the adsorption models, we used an 8 × 8 supercell of a pristine SnS_2_ slab, as described in detail below. The study of the adsorption energy trends over different supercell sizes shows that this cell size is sufficiently large to prevent lateral interactions between the adsorbed metal atoms in different replicas (Table S10). After atomic relaxation, all adsorption models retained their symmetry.

### Electrochemical CO_2_RR Performance

In our study we evaluated the electrochemical performance of CO_2_RR toward formic acid production using the developed models for SACs and DACs. As a descriptor for the efficiency and selectivity of the CO_2_RR on the different catalysts, we used the electrochemical *U*_*L*_ for each reaction pathway. However, when the highest energy barrier was associated to a nonelectrochemical step, we used the associated thermodynamic barrier instead. The selectivity between formic acid and other potential products was decided by comparing the highest thermodynamic barrier for all reaction pathways on each catalyst. The evaluation of the Δ*G*_ads_ for reaction intermediates and *U*_*L*_ along the CO_2_RR pathways is detailed in the Computational Methods section.

**Figure 2:**
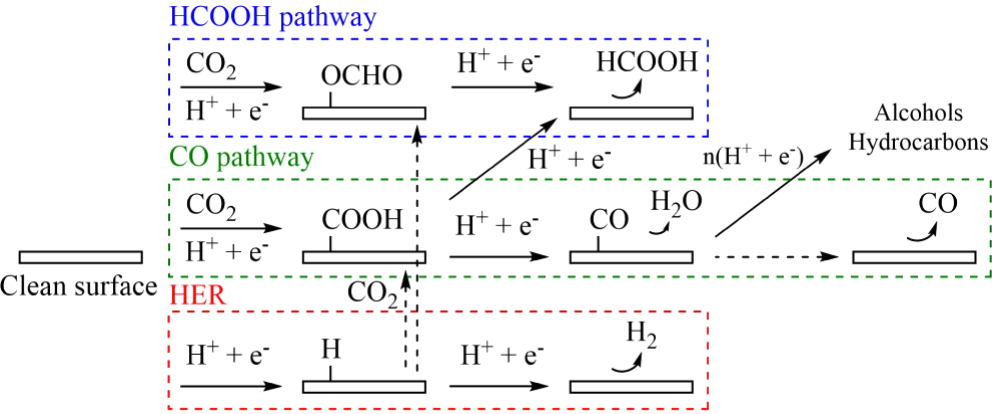
Schematic diagram of possible electrochemical pathways for the CO_2_ reduction reaction and hydrogen evolution reaction on SACs and DACs. The solid and dashed arrows represent electrochemical and nonelectrochemical steps, respectively.

According to the established mechanism for CO_2_RR,^47^ the first electrochemical step can proceed in two directions. One pathway leads to the production of formic acid (HCOOH) via a two-electron reduction process, passing through a formate intermediate (*OCHO). The other pathway initially forms a carboxyl intermediate (*COOH), which is subsequently reduced to *CO, where * denotes adsorption over the metal center. The reactions stemming from the *CO intermediate are complex because the CO molecule can desorb from the surface directly or undergo further reduction into other products. In other studies, it was also proposed that *COOH could be possibly reduced to HCOOH^48,49^ (see Figure 2). The initial electrochemical steps in each pathway strongly influence the overall direction of the CO_2_RR. Therefore, to unravel the ability of our catalysts to produce formic acid, we simulated and compared two parallel reaction pathways: a complete two-step HCOOH pathway and a short CO pathway with a gas-phase CO molecule as the final product.

The route toward the formation of the first CO_2_RR intermediate can either involve a concerted electron–proton pair transfer step or a decoupled electron transfer process resulting in a radical *COO^–^ anion, chemically bonded over the catalyst surface, followed by proton transfer.^50^ The bonding of this anion to the surface through C or O would determine the progression of CO_2_RR toward CO or HCOOH, respectively,^51^ and the reaction product would depend on the *COO^–^ adsorption energy.^52^ Actually, the intrinsic redox potential for CO_2_ reduction into COO^–^ is generally high,^53^ making this pathway unlikely under typical conditions. However, the catalytic activity of our SACs and DACs could potentially make this reduction process energetically viable; therefore, we tested this hypothesis in our investigation and also studied the competition between CO_2_ and H_2_O adsorption.

Finally, a key challenge in electrochemical CO_2_RR is its competition with the hydrogen evolution reaction (HER). For a catalyst to be effective, it must exhibit selectivity toward the desired reduction products over HER. Therefore, we also studied the effects of HER on our catalysts and compared its outcomes with those of CO_2_RR. In addition, for completeness, we considered the potential pathways where CO_2_ reacts with adsorbed *H to form *OCHO or *COOH (see Figure 2).

#### Structure of the Catalyst Surface: Pourbaix Diagrams

**Figure 3:**
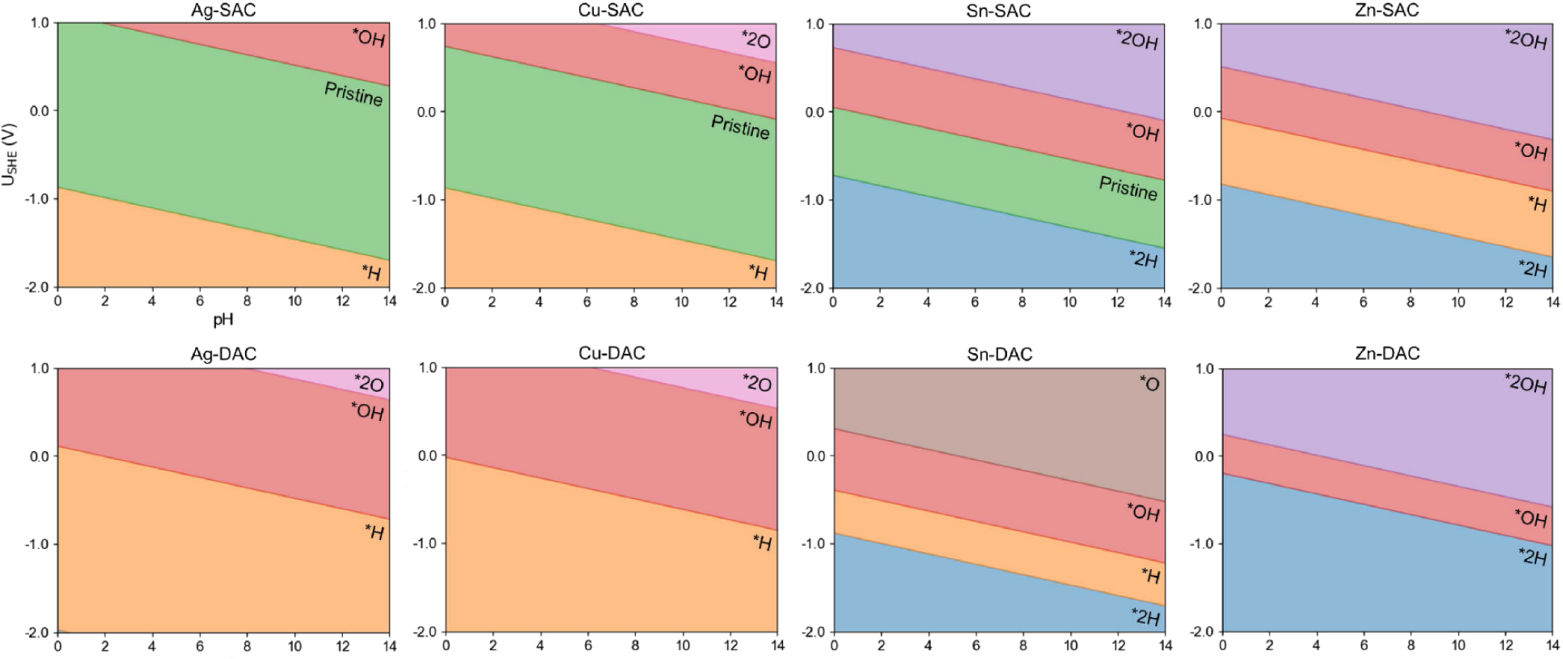
Pourbaix diagrams for the studied catalyst surfaces under different potentials and pHs.

Before evaluating the energetics of the electrochemical CO_2_RR, we investigated the equilibrium configurations for the proposed catalyst surfaces. Specifically, the metal sites on SACs and DACs may be covered by *H at the negative potentials typically applied during CO_2_RR,^54^ which may result in the competing HER and thus lower CO_2_RR selectivity. We calculated the Pourbaix diagrams for the adsorption of *OH, *O, and *H on the catalyst surfaces. As shown in Figure 3, the M-SACs with M = Ag, Cu, and Sn exhibit a wide potential window for the stability of clean metal-decorated surfaces. The adsorption of *H on these catalysts occurs at potentials lower than their CO_2_RR operating potentials; therefore, HER is less likely to happen. For Zn-SAC, Ag-DAC and Cu-DAC, *H covers available metal sites. As a result, the evaluation of the electrochemical CO_2_RR on these catalysts will start with hydrogenated surfaces. The presence of *H can either lead to atypical CO_2_RR reaction pathways or directly to the HER, depending on the adsorption energy of the CO_2_RR intermediates. On Sn-DAC and Zn-DAC at negative potentials, the surface can be either covered with *OH or *H, depending on the potential range. The possible reactions occurring on these surfaces are detailed in the paragraph below.

#### Adsorption Free Energy Pathways for CO_2_RR

Our analysis of the reaction pathways for CO_2_RR revealed that Sn-SAC was the most efficient catalyst for producing formic acid, exhibiting the lowest electrochemical *U_L_* of −0.29 V (see Table 1). The significantly higher *U*_*L*_ for the CO pathway on Sn-SAC indicates its high selectivity toward formic acid production. While other catalysts may have a smaller *U_L_* in CO_2_RR compared to Sn-SAC, their *U*_*L*_ for HER are comparable or smaller (see Table S9). As a result, the competitive HER on other surfaces is much more viable, making them not suitable for selective electrochemical CO_2_RR. In our knowledge, the *U*_*L*_ on Sn-SAC is comparable to the smallest *U*_*L*_ reported in theoretical studies for other materials known to selectively produce formic acid,^48,49,55,56^ as well as other SACs and DACs materials non-necessarily selective to formic acid^44,57−61^ (see Table S7). We therefore propose Sn-SAC as a promising candidate for efficient and selective formic acid production in electrochemical CO_2_RR.

**Table 1:** Limiting Potentials of Different CO_2_RR Pathways on Each Catalyst^a^

Reaction pathway	CO_2_ → HCOOH (V)		CO_2_ → *CO (V)	
System	SAC	DAC	SAC	DAC
Ag	–1.33	–0.40	–1.65	0 (0.60 eV)
Cu	–1.26	–0.76	–1.66	0 (0.19 eV)
Sn	–0.29	–0.40	–1.52	–1.10
Zn	–0.30	–0.19 (0.21 eV)	0 (0.66 eV)	No opening pathway

a When the maximum energy barrier along the CO_2_ reduction pathway is associated to a nonelectrochemical step over the catalyst, the energy barrier is reported in parentheses.

When studying CO_2_ adsorption on the catalyst surfaces, we first addressed the question whether it is adsorbed in a neutral-state *CO_2_ or as a *COO^–^ radical. We found that on all catalysts, CO_2_ binds as a neutral molecule to the metal centers through one of its O atoms on the SACs, and with two O atoms symmetrically adsorbed on the DACs (see Figure S2). Moreover, on all surfaces, adsorbed *CO_2_ remained linear. Notably, for Sn-SAC and Sn-DAC, the distances between CO_2_ and metal atoms were larger (see Table S6). To the best of our ability, we could not find any metastable configuration for the radical *COO^–^ anion over the surface. Thus, this process is expected to have a high-energy barrier. Based on these observations, CO_2_ on SACs and DACs is more likely to be physisorbed or weakly chemisorbed in a neutral state rather than chemisorbed as a radical anion.

To determine whether the first step of CO_2_RR on the catalysts involves the adsorption of neutral CO_2_, we then compared the adsorption free energies of CO_2_ () and H_2_O () on the metal centers. We found that M-SACs and M-DACs with M = Ag, Cu, Zn all have negative  and positive  (see Table 2), thus the H_2_O molecules bind to the metal centers instead of CO_2_ on these surfaces. On Sn-SAC and Sn-DAC, the  and  are both positive. However,  is small and also much lower than , which indicates that adsorbing H_2_O is more favorable than CO_2_. As a result, CO_2_RR on Sn-SAC likely occurs through a concerted mechanism where a proton from the solution transfers to the CO_2_ molecule in solution as it approaches the metal site to give *OCHO chemisorbed on the clean catalyst surface, as suggested by most literature.^44,48,49,55,56,60,61^ It is worth noting that the free energy of solution for CO_2_ in water is estimated to be 0.19 eV based on experimental data,^62^ which implies that the free energy state of CO_2_ in the aqueous-solution phase is higher than that in the gas phase, and the adsorption of it onto the surfaces may actually require less energy, although the reduced  is still larger than .

**Table 2:** Adsorption Free Energies of CO_2_ and H_2_O on Metal Sites of Catalysts

System	SAC (eV)		DAC (eV)	
				
Ag	0.33	–0.32	0.29	–0.34
Cu	0.25	–0.50	0.23	–0.30
Sn	0.52	0.17	0.47	0.02
Zn	0.25	–0.64	0.35	–0.45

**Figure 4:**
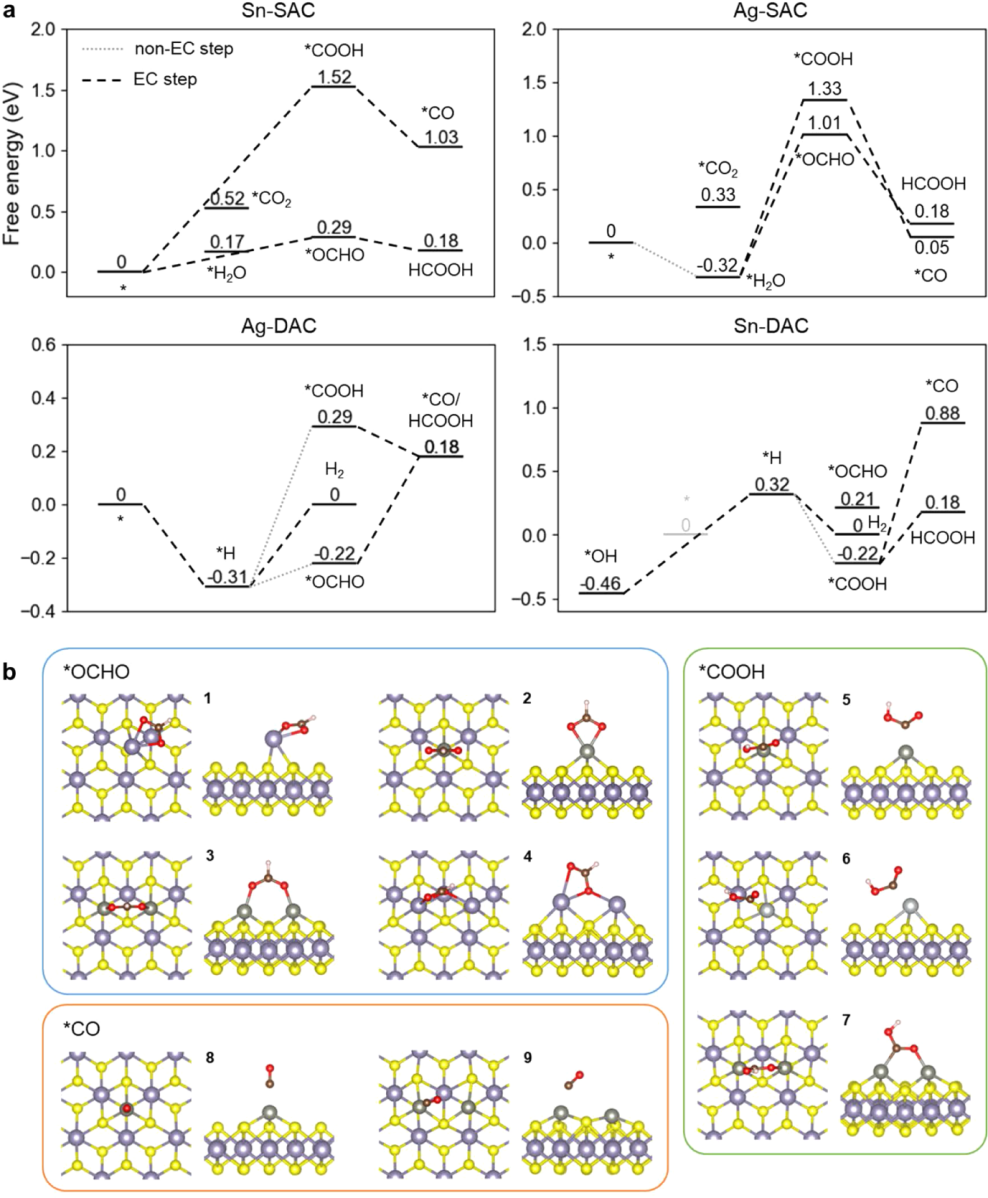
(a) Free energy profiles of potentially favorable reaction pathways toward the formation of formic acid, *CO, and H_2_ on SACs and DACs with Ag and Sn. EC stands for “electrochemical” in the legend. (b) Selected configurations for CO_2_RR intermediates adsorbed on the surfaces of SACs and DACs. Yellow, purple, gray, light-gray, red, brown, and white balls represent S, Sn, Zn, Ag, O, C and H atoms, respectively.

The detailed reaction free energy profiles on each catalyst are depicted in Figures 4 and S3.

Notably, the *U*_*L*_ of −0.29 V for CO_2_RR on Sn-SAC can be an overestimation of the potential-limiting barrier. Since the  barrier of 0.17 eV can be easily overcome at room temperature,^45^ it is possible that CO_2_RR starts from a hydrated surface. In such case, after overcoming a nonelectrochemical barrier of 0.17 eV, it takes only −0.12 V to make the reaction proceed. Therefore, the two reaction pathways can see *OCHO either directly adsorb onto the pristine surface or displace adsorbed water, depending on the activation barrier for these processes. The barrier for proton transfer from the solution to CO_2_ is expected to be low due to CO_2_’s solvation in aqueous phase, which forms hydrogen bonds with surrounding water molecules and possible hydronium ions. This direct pathway may have a lower activation barrier compared with a pathway where H_2_O adsorbs over the surface and is then displaced by *OCHO.

On Ag-SACs and Cu-SACs, the initial electrochemical step starts with H_2_O adsorbed on the surface. However, as the adsorption energies of *OCHO and *COOH are both high, large limiting potentials characterize both pathways on the catalysts; thus, electrochemical CO_2_RR can hardly proceed. For most of the other systems, instead of adsorbing water, the CO_2_RR reaction proceeds starting from *H, which is then transferred to CO_2_ via a nonelectrochemical step to give *OCHO or *COOH. On Zn-DAC, where *2H is adsorbed, the addition of a CO_2_ molecule via a nonelectrochemical step may directly give HCOOH.

With *H initially adsorbed on the surfaces, Zn-SAC, Ag-DAC, and Cu-DAC systems face a problem of competitive HER. For example, as shown in Figure 4, on Ag-DAC the nonelectrochemical thermodynamic barrier from *H to *OCHO is only 0.09 eV, and the *U*_*L*_ for the reduction from *OCHO to formic acid is −0.40 V, a relatively small value. However, HER can occur with a smaller *U_L_* of −0.31 V. When an increasingly negative potential is applied, the nonelectrochemical barrier is not affected, while the rise in free energy along the HER pathway is gradually reduced to zero, making the reaction free energy difference between CO_2_RR and HER even larger. As a result, these catalysts are likely to show selectivity to HER instead of CO_2_RR.

On Sn-DAC, *OH and *H coverages are at equilibrium at *U* = −0.39 V. This was the smallest potential required to reduce *OH to *H and open up possible CO_2_RR pathways. The reaction proceeds with the combination of CO_2_ and *H to give *COOH. The next step gives formic acid, and this step is associated with a *U_L_* of −0.40 V. However, the *U*_*L*_ for HER is only −0.32 V, which means HER already proceeds spontaneously at *U* = −0.39 V. As a result, Sn-DAC can be more selective toward HER and is not promising for CO_2_RR. The same issue was also found on Zn-DAC, despite *2H was preferably adsorbed rather than *H.

In summary, among the proposed catalysts, we found that Sn-SAC displays both good activity and high selectivity toward the production of formic acid for electrochemical CO_2_RR, with a small *U*_*L*_ of only −0.29 V. Ag-SAC and Cu-SAC suffer from large limiting potentials along CO_2_RR pathways, while the rest show comparable efficiency in producing H_2_ as well as CO_2_RR products, resulting in poor selectivity toward the desired reactions.

The geometric structures of relevant reaction intermediates are depicted in Figure 4 and are described more in detail in the following.

For the key intermediate *OCHO along the HCOOH pathway, inset **1** shows that *OCHO is adsorbed on Sn-SAC in a tilted configuration, which is characterized by a lower adsorption free energy compared to a vertical configuration. Conversely, the vertical configuration of *OCHO, as shown in inset **2** of the Zn-SAC as an example, is more stable on other SACs than the tilted one. The comparison between SACs and DACs shows that, for most DACs, the adsorption free energy of the *OCHO intermediate (ΔG_*OCHO_) is reduced compared to SACs with the same metal type (see Table S8). By comparing the configurations in the insets of **3** and **2**, it can be seen that the reason for the enhanced stability of this intermediate is the extra bond formed between the adsorbed moiety and the second metallic site, resulting in a bidentate chemisorption configuration. A notable exception is Sn-DAC, shown in the inset of **4**, where the most stable configuration for *OCHO connects only one O atom to both Sn active sites. This unique connection leads to a slight stabilization of *OCHO in Sn-DAC than Sn-SAC. The final step along this pathway is the release of HCOOH following another proton transfer from the environment. The free energy for the adsorption of *OCHO on both Sn-SAC and Sn-DAC is close to that required to release a free HCOOH molecule, showing that desorption from the surface should occur easily.

Another key intermediate, *COOH, is relatively unstable for all SACs except for Zn-SAC. On Zn-SAC, *COOH binds to the metal center with its C atom positioned vertically, as shown in inset **5**. This is in contrast to other SACs, where SACs tilts toward the basal sulfur plane on the OH side, making this configuration less favorable (inset **6**). The comparison between SACs and DACs shows that ΔG_*COOH_ is lower for DACs compared to SACs, similar to the HCOOH pathway. The comparison of the configurations in insets **5** and **7** shows that stable bidentate configurations were also observed on all DACs for *COOH. The next step along this pathway follows another electron–proton pair transfer and the release of a water molecule, which leads to a *CO molecule being adsorbed vertically on metallic sites via its C end, as shown in inset **8** for Zn-SAC, with a similar configuration for each catalyst. The behaviors of *CO on SACs and DACs are similar; therefore,  is only slightly higher on DACs than on SACs with the same metal type. This also confirms the essential role of the second bonds between intermediates and dual-atom sites, as *CO does not bond with the dual-atom site through the O atom (inset **9**).

Finally we compared the activity of the SACs and DACs with that of the pristine substrate SnS_2_. The analysis of the free energy changes for CO_2_RR on the SnS_2_ monolayer shows that ΔG_*OCHO_ and ΔG_*COOH_ are 3.35 and 2.02 eV, respectively, much higher than those on any SAC or DAC surface. This proves that the adsorption of metal atoms on the SnS_2_ surface forms essential catalytic centers to stabilize the intermediates and facilitate the reactions.

### Catalyst Stability

Since the stability of SACs and DACs is an important concern, we evaluated the formation energy (*E*_Form_) for the metal atoms over the pristine SnS_2_ monolayer:
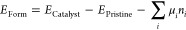
5where *E*_Catalyst_ is the total energy of the decorated monolayer, *E*_Pristine_ is the total energy of the pristine SnS_2_ monolayer, and μ_*i*_ and *n*_*i*_ are the chemical potential and number of metal atoms of type *i* adsorbed over the pristine SnS_2_ structure. The chemical potential of each element was evaluated as the total energy per atom in its elemental form, i.e., in the bulk for metals. A high *E*_Form_ indicates that the formation of the catalyst is more difficult, while a low *E*_Form_ means that the catalyst is more easily formed. The PBE-D3 functional was used for these calculations because we found that the HSE-D3 adsorption energetics trends could be reproduced with PBE-D3 (see Table S8). By contrast, the HSE functional is less adequate and computationally more demanding for pure metal systems.

The calculated *E*_Form_ for each catalyst under low coverage conditions is reported in Table 3. At this coverage, the structures of Zn-SAC and Zn-DAC have the lowest *E*_Form_ among all of the catalysts (below 0.22 eV per atom). Sn-SAC and Sn-DAC also show relatively low *E*_Form_ values of approximately 0.28 and 0.37 eV, respectively. The low *E*_Form_ values for these catalyst candidates suggest that their experimental synthesis is relatively straightforward. In contrast, the *E*_Form_ values for Ag-DAC and Cu-SAC were higher. Although these catalysts are both efficient, their preparation may be more challenging.

**Table 3:** Formation Energy Per Atom for SACs Adsorption Models at Low Coverage

Metal	SAC (eV)	DAC (eV)
Ag	0.487	0.506
Cu	0.521	0.503
Sn	0.284	0.372
Zn	0.214	0.167

We also evaluated the *E*_Form_ for substitution models,^25^ in which the decorating metal atom replaced an in-plane Sn atom in pristine SnS_2_ (see Figure S4). However, these substitution models generally exhibit much higher *E*_Form_ than the adsorption models with the same metal (see Table S11), which means that they are less stable and therefore are not further discussed in this work.

In the experimental preparation of SACs and DACs with distinct catalytic sites, it is crucial to prevent the agglomeration of metal atoms. To assess the likelihood of this occurrence in our proposed adsorption models, we explored how *E*_Form_ varies as a function of decorating metal atom coverage on the SnS_2_ substrate. For the analysis of SAC systems, we generated models with different supercell sizes (2 to 10 × 10), each with a single metal atom adsorbed. To model coverage above 0.25 ML, we increased the number of metal atoms (two to four) on a 2 × 2 supercell.

**Figure 5:**
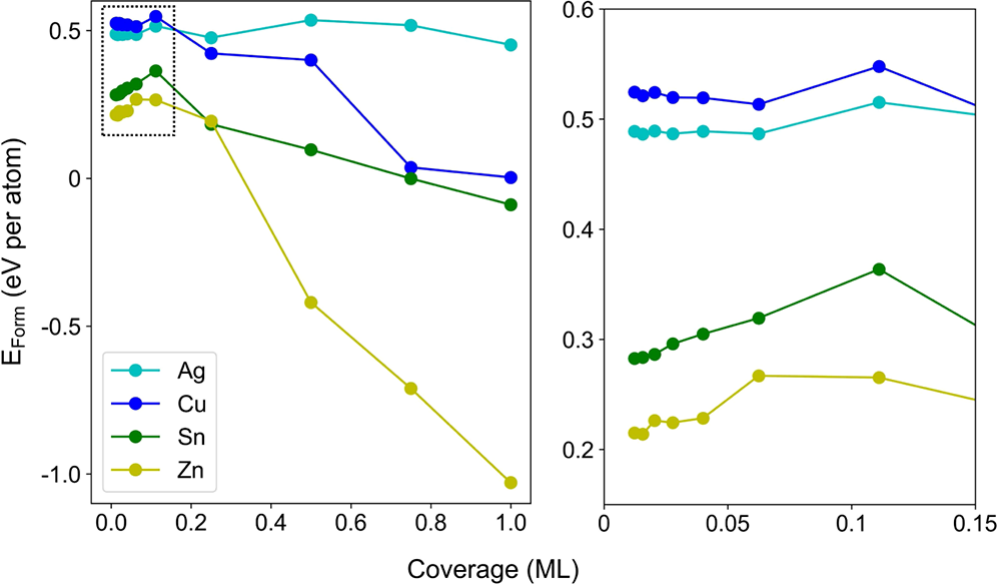
Left: formation energy trends for SACs models as a function of the coverage of adsorbed metal atoms. Right: zoom-in graph of the low coverage area enclosed within the dotted box.

As shown in Figure 5, when coverage is below 0.10 ML, both Ag-SAC and Cu-SAC have a metastable state near 0.06 ML. For Sn-SAC and Zn-SAC, *E*_Form_ tends to decrease as coverage decreases, but the rate of change diminishes gradually, indicating potential metastable states at sufficiently low coverage. For coverage above 0.10 ML, Ag-SAC exhibited another metastable state at 0.25 ML, with a slightly lower *E*_Form_ than that at 0.06 ML. *E*_Form_ for the other systems rapidly decreased as the coverage increased above 0.10 ML. This indicates a higher tendency of these metals to aggregate on the SnS_2_ monolayer.

Similar trends in *E*_Form_ were also observed for DACs at low coverage, with two metal atoms adsorbed on the SnS_2_ substrate in adjacent positions (see Figure S5). These calculations suggest that it is possible to prepare SACs and DACs on SnS_2_ monolayers with low metal loads to prevent clustering. In particular, for the selective Sn-SAC catalyst, there is an energy barrier of about 0.08 eV to overcome before agglomeration occurs.

### Charges and Electronic Structures

To understand the connection between the adsorption energies of each single adsorbate and their electronic properties, we conducted electronic structure calculations and Bader charge analysis^63−66^ on each system. We determined the charge of each atom by subtracting its calculated Bader charge from its valence electron charge. For the pristine SnS_2_, we observed a uniform charge distribution characterized by negatively charged sulfur planes and positively charged Sn plane. Specifically, each S atom carried a charge of −2 |*e*|, and each Sn atom carried a charge of +4 |*e*|.

**Figure 6:**
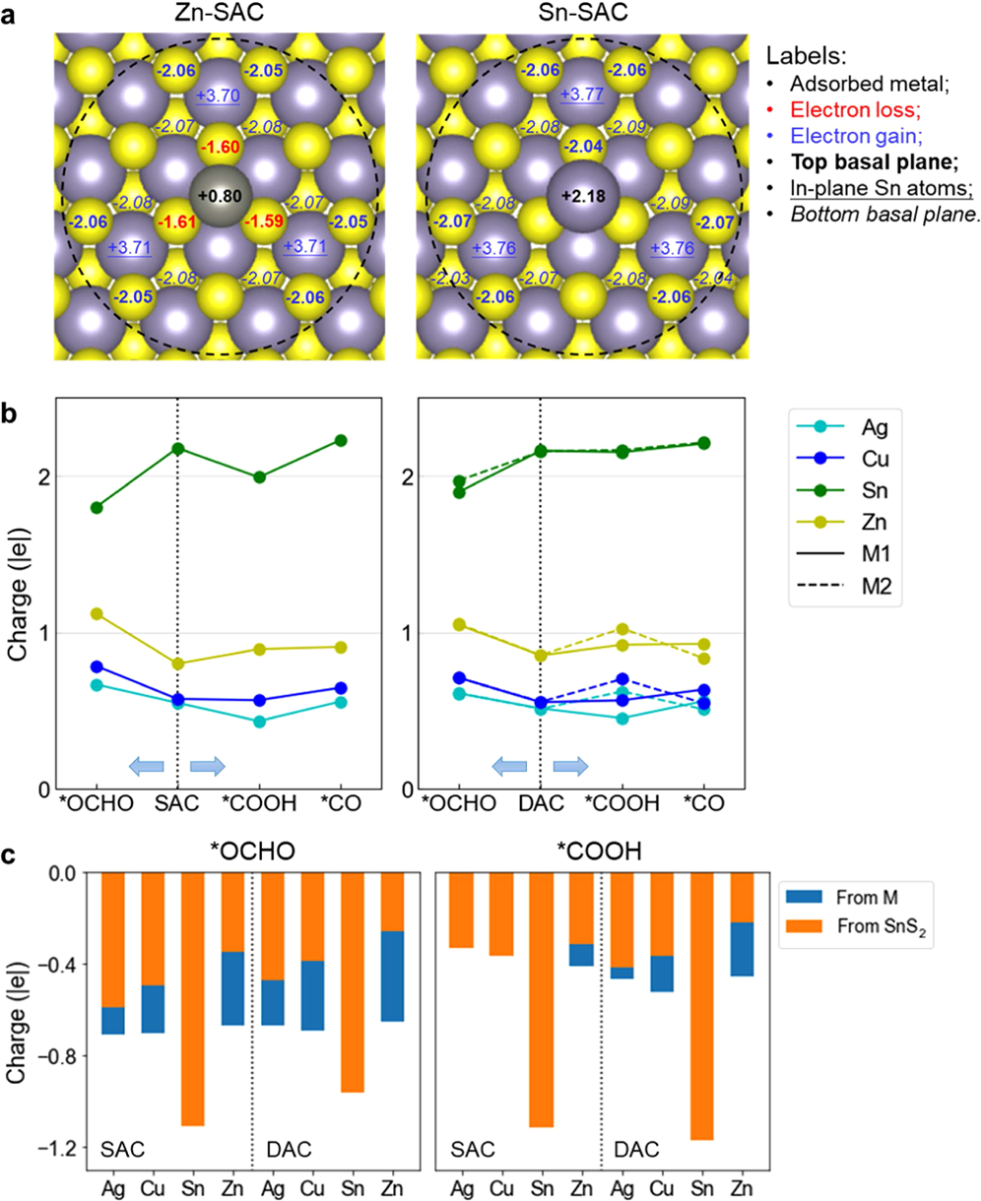
(a) Atomic charges on Zn-SAC and Sn-SAC. The values of atomic charge are calculated with the number of valence electron subtracting calculated Bader charge (|*e*|). Only atoms showing charge variation above 0.03 |*e*| compared to pristine SnS_2_ are labeled. (b) Charges and their variations on adsorbed metal atoms in CO_2_RR. For DACs, M1 and M2 represent the atoms connected to C and O of *COOH, respectively. (c) Total charge gains of *OCHO and *COOH moieties adsorbed on SACs and DACs.

Since our preferred catalyst is Sn-SAC, we mainly focused our analysis on SACs systems. The analysis of the charges on these systems shows that the adsorption of the metal atoms induces charge redistribution, which extends to a radius of about 6.5 Å around the metal sites, as illustrated in Figure 6a.

More specifically, in Sn-SAC, the adsorbed Sn atom carries a significantly positive charge of +2.2 |*e*|, which is higher than that of the other metal centers. Additionally, the S atoms nearest the Sn atom are slightly more negatively charged. However, the total charge of the Sn atom combined with the adjacent S atoms is approximately +2 |*e*| in this case. In this system, the positive charge is concentrated at the metal center, resulting in the Sn atom likely acquiring an oxidation state of +2. The two electrons are transferred to the next shell of atoms, which is overall negative, with Sn and S atoms more negatively charged than the pristine SnS_2_ by approximately 0.07 |*e*| and 0.24 |*e*| per atom, respectively. It has been reported that Sn surface doped with subsurface S atoms creates more positively charged Sn centers that have higher affinity to *OCHO rather than *H.^67^ The preferred trend toward CO_2_RR over the HER on Sn-SAC can also be attributed to the similar configuration and charge pattern over the surface.

In the case of the Zn-SAC system, the adsorbed metal atom becomes positively charged by approximately +0.8 |*e*|, and the S atoms next to it become more positive than those in pristine SnS_2_ (each S atom loses about 0.4 |*e*|, see Figure 6a). This results in the formation of a positively charged center with an overall charge of around 2 |*e*|. Therefore, although the calculated atomic charge on Zn is approximately +1 |*e*| due to inaccuracies in charge evaluation from the Bader scheme combined with a small charge delocalization around the metal center, this might also be consistent with an oxidation state of +2 for Zn.^68^ The next shell of Sn and S atoms becomes more negative by approximately 0.3 |*e*| per Sn atom and 0.06 |*e*| per S atom, respectively. In the cases of Ag-SAC and Cu-SAC, the metal center is positively charged by 0.5 to 0.6 |*e*| (see Figure S6). In these systems, the next S atoms acquire a positive charge of approximately 0.1 |*e*| each, with an overall charge of the metal atom combined with the adjacent S atoms of approximately 1 |*e*|. Thus, the oxidation states for Ag and Cu over the substrate would be +1.

The analysis of the charge redistribution can also help to rationalize the differences in the stability of differently decorated SACs. Indeed, the electrostatic repulsion among the more positive Sn-SAC and Zn-SAC catalytic centers stabilized the low coverage in these configurations. For Ag-SACs and Cu-SACs, the minimum cell size to represent nonoverlapping circles is 4 × 4, which was used to model the 0.06 ML coverage for Ag-SACs and Cu-SACs (Figure 5). In such systems, the positive charges of the metal centers are gradually screened by the neighboring S atoms, eventually reaching a neutral region beyond the boundary of the charge redistribution areas. Thus, while the system is destabilized when the charged regions start overlapping at 0.06 ML, this is also the coverage where the electrostatic interaction between positive and negative shells is maximized, hence the minimum in the formation energy profile.

**Figure 7:**
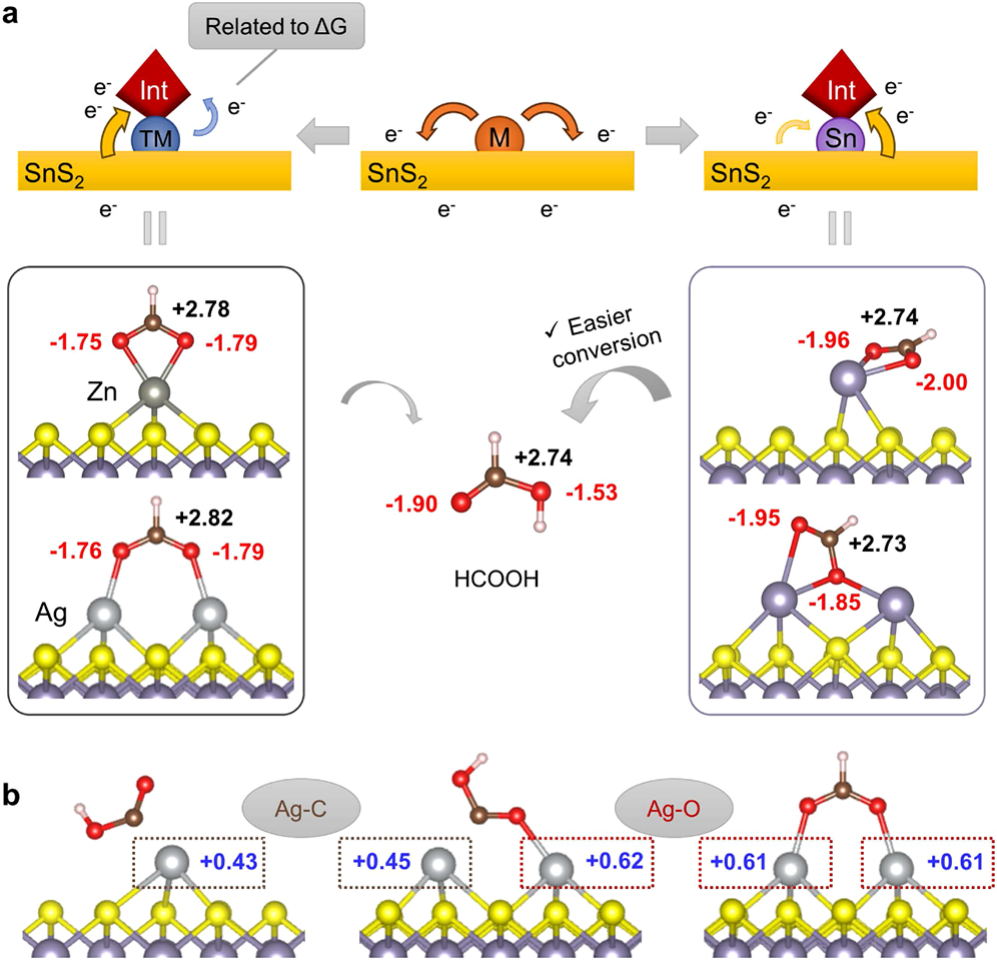
(a) Schematic diagram of electron transfer on the clean surfaces and the surfaces adsorbing intermediates. The size and color of the arrows indicate the amount and source of electron flow. The models show atomic charges on *OCHO adsorbed on various SACs and DACs, compared with the charges on isolated formic acid. (b) Atomic charges on Ag atoms linked to the same atoms in different intermediates. The black, red, and blue numbers represent charges on C, and O and metal atoms, respectively.

The charge redistribution during CO_2_RR is shown for all systems in Figure 6b. Interestingly, when the *OCHO intermediate is formed, we observed electron losses on metal centers except Sn, which have the opposite direction of charge transfer. And when *COOH is formed, we observe electron gains on the SAC metal centers except Zn, but electron losses on the DAC metal centers except Ag. By analyzing the total charges on the adsorbates (Figure 6c), we found that the electron gains on intermediates were much higher than the charge variations on the metal centers. In fact, the SnS_2_ substrate plays a major role in donating electrons to intermediates. The electrons are transferred from the polarized metal centers to the substrate when metal atoms are adsorbed and are partially released into intermediates when CO_2_RR proceeds (Figure 7a). In some systems, especially for Sn-SAC and Sn-DAC, the metal centers also receive negative charges donated back from the substrate. In both cases, the metal centers may act as the mediators of charge transfer in these processes because no bond forms between intermediates and the electron-rich basal sulfur plane.

Since the number of electrons gained by *OCHO was very similar for all the catalysts except Sn-SAC and Sn-DAC, this should not be a strongly relevant descriptor to the binding strength of intermediates. It is more likely that net charge transfer from metal centers to intermediates is associated with the adsorption energy: when *OCHO is adsorbed on SACs, the electron loss for the metal center is highest on Zn (0.32 |*e*|) and lowest on Ag (0.12 |*e*|), which has an inverse trend compared to their ΔG_*OCHO_. The charge variations are smaller for *COOH because Zn loses 0.09 |*e*| and Cu has almost unchanged charge, while Ag gains 0.12 |*e*|. As a result, their ΔG_*COOH_ values are generally higher than those of ΔG_*OCHO_. Therefore, it is possible that a higher net charge transfer from the metal to the intermediate corresponds to lower Δ*G*_ads_.

The mechanism for Sn-SAC and Sn-DAC may be different, as the charge transfer is stronger between the substrate and adsorbates, but they do not show overly high or low ΔG_*OCHO_. We analyzed the atomic charges of *OCHO on different surfaces. As more electrons are donated to *OCHO on Sn-SAC and Sn-DAC, the C and O atoms are actually more negative, which leads to similar charge distributions between *OCHO and the isolated HCOOH molecule (Figure 7a). This may result in close free energies between *OCHO and HCOOH; therefore, protonation of *OCHO can occur with a small free energy change leading to low U_L_ in overall CO_2_RR. More negative O atoms may also facilitate the protonation step.

Moreover, we found that the behavior of each metal atom on DACs resembled that on SACs. The calculated charge loss to *OCHO of each metal atom on DACs is similar to that on SACs with the same metal (Figure 6b), which means nearly double number of electrons are being donated from metals to *OCHO on DACs compared to SACs. Correspondingly, greatly reduced ΔG_*OCHO_ values were observed. And when *COOH is adsorbed on DACs, the metal atom connected to C (M1) has very similar charge transfer to the one in SACs, and the other metal atom connected to the O atom (M2) shows similar charge variations compared to metal atoms adsorbing *OCHO through O atoms (Figure 6b). An example of a detailed comparison of charges on Ag-decorated catalysts is provided in Figure 7b. With the M2 site donating more electrons to *COOH through the extra M-O bond, the carbon is reduced to a more negative state and ΔG_*COOH_ decrease significantly on DACs compared to SACs. Based on these understandings, it implies that the behaviors of the two metal atoms on DACs are quite independent. Therefore, it is possible to design combinations of dual-atom pairs on DACs to modulate the charge transfer from catalytic centers to intermediates and thus engineer catalysts with optimized adsorption energy of key intermediates in electrochemical reactions.

**Figure 8:**
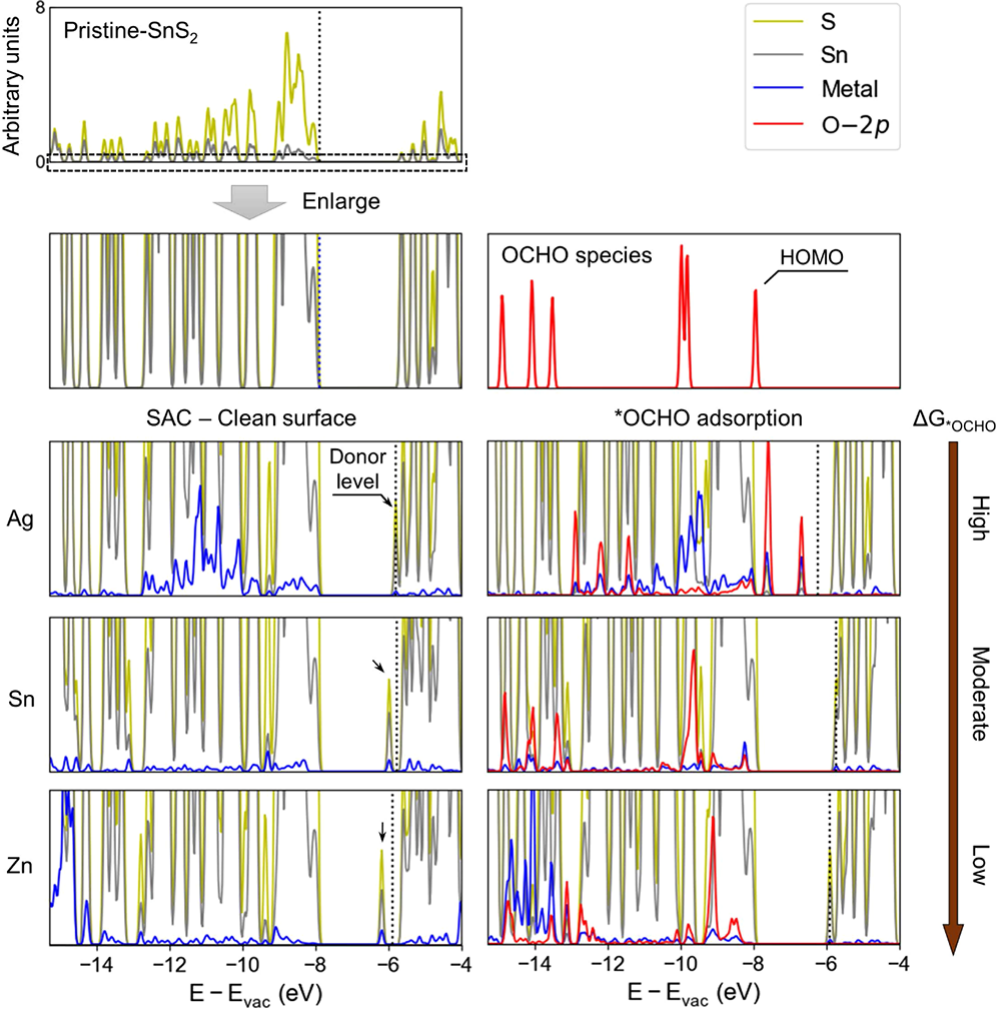
PDOS of pristine SnS_2_ monolayer, isolated OCHO species, clean SAC surfaces, and surfaces adsorbing *OCHO, ranked in order of descending ΔG_*OCHO_. The enlarged bottom parts (within the area of the dotted box) of PDOS are shown for details. Energies are aligned to the vacuum level. The black dotted line represents the Fermi energy.

We also investigated the projected density of states (PDOS) of the catalysts (Figures 8 and S7). The adsorption of metal atoms creates new occupied energy levels near the original conduction band minimum of SnS_2_, similar to an electron donor band in a doped semiconductor. As strong electron-donating abilities can promote the first electrochemical reduction step of CO_2_,^69^ this may account for the higher CO_2_RR activity of our catalysts compared to the inert pristine SnS_2_. Yet these donor levels are mainly composed of orbitals from S and Sn atoms, the SnS_2_ substrate still plays an important role in providing electrons in the reactions, and the small contributions of the adsorbed metal atoms to the donor levels indicate their role as merely mediators, which are consistent with the Bader charge analysis.

Within the range of valence band, Ag and Cu atoms show strong densities below the valence band maximum, while the majority of PDOS on the Zn atom is located in deeper energy range. Particularly, the adsorbed Sn on the surfaces shows much lower densities across the valence band, which may result from fewer valence electrons than TMs. Indeed, Sn-SAC and Sn-DAC both show unique performances compared to other catalysts with TM centers: the adsorption configurations of *OCHO and *COOH on them are distinct, and they have similarly moderate binding strengths to *OCHO but destabilized adsorption of *COOH or *CO, as also observed in other studies.^56,70^ This implies that other *p*-block metals may demonstrate the same features in electrochemical CO_2_RR due to similar valence electron configurations, and therefore, are also appealing in fabricating SnS_2_ supported catalysts for selective formic acid production.

When *OCHO is adsorbed on Ag-SAC, we observed that PDOS on *OCHO shifts toward higher energy and creates new hybridized donor levels within the band gap, which may account for its high positive adsorption free energy of *OCHO. On Sn-SAC and Zn-SAC, mixing between PDOS on metal atoms and *OCHO occurs at lower energy levels that have slight shifts compared to PDOS of isolated OCHO species. Interestingly, Ag-DAC and Sn-DAC show similar energy ranges of these hybridized densities (see Figure S7). Given that these catalysts all have moderate ΔG_*OCHO_, hybridization between PDOS of catalysts and *OCHO within this range can be a sign of suitable catalysts for efficient CO_2_ reduction to formic acid. By contrast, as the hybridized PDOS shifts toward lower energy levels on Zn-DAC (see Figure S7), it shows strongest adsorption of *OCHO; therefore, hindering further reduction.

## Conclusions

In this study, we constructed SACs and DACs for electrochemical CO_2_RR based on the SnS_2_ monolayer substrate, with Ag, Cu, Zn, and Sn metal atoms adsorbed on its surface as the catalytic centers. Evaluations of the stable surface state via Pourbaix diagrams show that only M-SACs with M = Ag, Cu, and Sn have available stable pristine surfaces, while the metal sites on the other catalysts are covered with *H. The generally higher adsorption free energy of CO_2_ than H_2_O on the surface implies that adsorbed *CO_2_ may not be involved in CO_2_RR pathways. The initial reduction step can therefore occur via a concerted mechanism, with hydrogen from the solvent directly transferred to CO_2_ and the formation of a reduced intermediate. Among the catalysts with an available pristine surface, Sn-SAC was found to be efficient in selective formic acid production with a low *U*_*L*_ of −0.29 V. Notably, this *U_L_* for CO_2_RR on Sn-SAC is an overestimation of the potential-limiting barrier. Starting from the hydrated surface, after overcoming a nonelectrochemical barrier for water adsorption of 0.17 eV, the reaction proceeds with only −0.12 V. Bidentate binding configurations between intermediates *OCHO and *COOH and metal centers were generally observed on DACs, and the extra bonding was responsible for their lower adsorption energy on DACs.

The study of the catalyst stability showed that the formation energy of all catalysts was low, and Sn-SAC (with the best performance) was predicted to be synthesized with a low coverage of metal adsorbates. Bader charge analysis revealed that the adsorption of metal atoms induces charge redistribution on the substrate near the adsorption site, and charge transfer between catalysts and intermediates in the reactions is mostly provided by the electron-rich SnS_2_ substrate, while metal centers only act as mediators. On TM centers, higher charge transfer from metal to intermediates is associated with their stronger binding and lower adsorption energy. The charge transfer on Sn-SACs and Sn-DACs is stronger, leading to close energy states between *OCHO and formic acid and easy conversion with low *U*_*L*_. It was also found that the bidentate binding configurations on DACs increases the charge transfer from metal to intermediates and thus reduces the adsorption free energy compared to SACs. Charge transfers are relatively independent for the two metal centers on DACs connected to different atoms on intermediate; therefore, a rational design of dual-atom sites based on the affinity of metals to their binding atoms is possible. Analysis of electronic structures suggests that the adsorption of metal atoms on the SnS_2_ basal sulfur plane creates new donor levels within its original band gap, which account for the catalysts’ greater ability to reduce CO_2_. Notably, adsorbed Sn atoms on the SnS_2_ monolayer displays lower PDOS than TMs, which may be associated with the good catalytic performances of Sn-SAC; therefore, we suggest that other *p*-block metals could also be considered for constructing such catalysts for selective formic acid conversion, while whether HER can be competitive requires additional evaluations.

## Supporting Information Available

Additional computational details and results on electrochemical CO_2_RR pathways, catalyst stability, charges, and electronic structure calculations (PDF)
