# Supplementary material for: Efficient and Selective Electrochemical CO2 to Formic Acid Conversion: A First-Principles Study of Single-Atom and Dual-Atom Catalysts on Tin Disulfide Monolayers
Source: J Phys Chem C Nanomater Interfaces. 2024 Sep 17;128(38):15861–72. doi: 10.1021/acs.jpcc.4c02283 (PMC11440595; doi:10.1021/acs.jpcc.4c02283)
Supplement: Supplementary file 1 — jp4c02283_si_001.pdf [file jp4c02283_si_001.pdf]

Supporting Information

Efficient and Selective Electrochemical CO_2 to Formic Acid Conversion: A First-Principles Study of Single-Atom and Dual-Atom Catalysts on Tin Disulfide Monolayers

Guanming Chen,[†,*] Margherita Buraschi,[†] Rashid Al-Heidous,[†] Satyanarayana Bonakala,[‡] Fedwa El-Mellouhi,[‡] and Clotilde S. Cucinotta[†,**]

[†]Department of Chemistry, and Thomas Young Centre, Imperial College London, White City Campus, W12 0BZ, London

[‡]Qatar Environment and Energy Research Institute, Hamad Bin Khalifa University, PoBox 34110, Doha, Qatar

*Correspondence: g.chen21@imperial.ac.uk

**Correspondence: c.cucinotta@imperial.ac.uk

Table of Contents

1. Computational details
2. Electrochemical CO_2RR
3. Catalyst stability
4. Charges and electronic structures
5. References

1. Computational details

The unit cell of 2H-SnS_2 was used to reproduce the properties of bulk SnS_2 for parameter tests. The MOLOPT basis set choices are: TZV2P for Sn; TZV2PX for C and S; TZVP for other elements (H, O, Ag, Cu and Zn). The auxiliary density matrix method (ADMM) was used to assist HSE level calculations within CP2K[1,2], with the ADMM basis set choices of: cFIT3 for H, C, O and S; cFIT6 for Sn; cFIT9 for Ag, Cu and Zn.

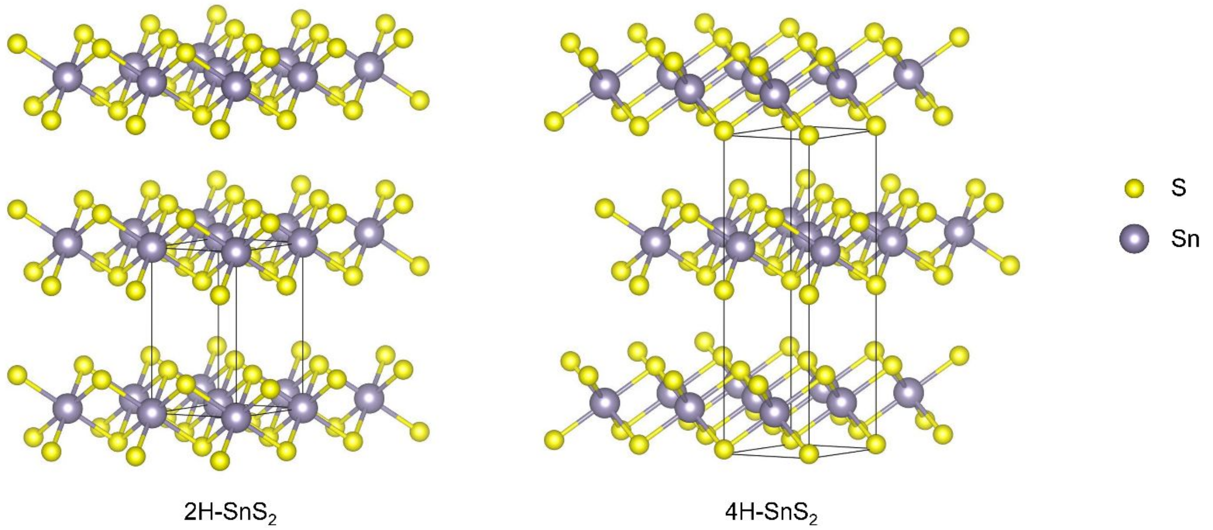

Figure S1. Structures of 2H- and 4H-polytype of SnS_2. The solid lines depict the unit cell of each polytype.

Table S1. Optimized lattice parameters of bulk-phase SnS_2

Level of theory	*a* (Å)	*c* (Å)
PBE-D3	3.70	5.69
HSE	3.71	6.01
HSE-D3	3.68	5.52
Experiment [3]	3.65	5.90

Table S2. Band gaps (ΔE_g) of SnS_2 bulk and monolayer based on structures at different levels

Level of geometry	Level of ΔE_g	Bulk (eV)	Monolayer (eV)
PBE-D3	PBE-D3	1.16	1.54
	HSE	2.07	2.52
HSE	HSE	2.31	2.53
HSE-D3	HSE-D3	1.91	2.53
Experiment		2.25 [3]	2.6 [4]

Table S3. Computational ΔE_g of SnS_2-related systems at levels of HSE-D3 with different screening parameters (ω)

Systems	HSE06-D3 (ω=0.11) (eV)	HSE-D3 (ω=0.08) (eV)	Reference (eV)
Bulk SnS_2	1.96	2.09	2.11 [5]
SnS_2 monolayer	2.33	2.48	2.52 [5]
S-vacancy on SnS_2 monolayer	0.653	0.774	0.75 [6]

Table S4. Calculated total energy, zero-point energy corrections, entropy contributions and Gibbs free energy of molecules at different levels of theory

	E_{Total} (eV)	ZPE (eV)	-TS (eV)	Free energy (G, eV)	G relative to references (eV)
PBE-D3					
H_2	-31.73	0.27	-0.42	-31.89	0
H_2O	-468.65	0.57	-0.55	-468.63	0
CO_2	-1027.83	0.32	-0.68	-1028.19	0
CO	-589.96	0.13	-0.61	-590.44	1.00
HCOOH	-1059.66	0.90	-0.96	-1059.45	0.35
HSE-D3					
H_2	-31.80	0.27	-0.42	-31.95	0
H_2O	-468.39	0.57	-0.55	-468.37	0
CO_2	-1026.50	0.33	-0.68	-1026.85	0
CO	-589.27	0.13	-0.61	-589.75	0.69
HCOOH	-1058.59	0.92	-0.96	-1058.43	0.18

To simulate formic acid in a dilute aqueous solution state under typical CO_2RR reaction condition, an approximation was implemented to treat HCOOH as an ideal gas with a fugacity of 2.0 Pa, which corresponds to an equilibrium between its gas and solution phase of 0.01 activity[8]. As the standard free energy change of reverse water-gas shift reaction is 0.30 eV, we found that calculated relative free energy of CO is overestimated to some extent with PBE-D3 and HSE-D3. This can be systematic computational errors for molecules in gas phase[8]. In comparison, HSE-D3 results are closer to experimental values in calculations of free energy of gas-phase molecules.

Table S5. Corrections on adsorption energy to simulate constant electrode potentials for electrochemical reactions

System		ΔE_{*OCHO} (eV)	ΔE_{*COOH} (eV)	ΔE_{*CO} (eV)
SAC	Ag	-0.16	-0.04	-0.05
	Cu	-0.12	-0.04	-0.04
	Sn	0.03	0.04	0.04
	Zn	0.00	0.01	-0.02
DAC	Ag	0.00	0.02	0.01
	Cu	-0.01	0.01	0.01
	Sn	0.01	0.07	0.08
	Zn	0.01	0.02	0.02

2. Electrochemical CO_2RR

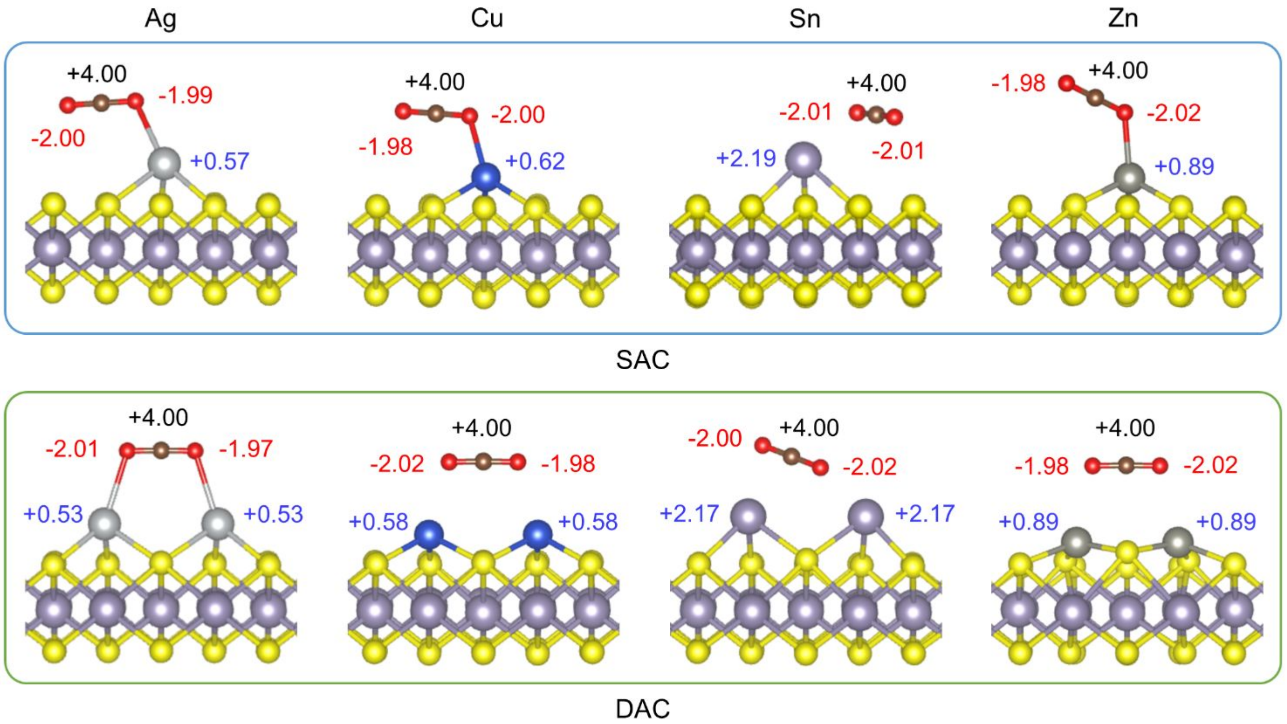

Figure S2. Adsorption configurations and Bader charges of CO_2 on SACs and DACs. Color of numbers represents: black, C; red, O; blue, metals.

Table S6. Adsorption free energy of CO_2 and distance between CO_2 and metal site on SACs and DACs

	System	Ag	Cu	Sn	Zn
M-O distance (Å)	SAC	2.52	2.27	3.47 (M-C)	2.29
	DAC	2.65	2.58	3.27 (M2-O)	2.73

Table S7. Comparisons of catalyst performances for electrochemical CO_2RR in computational work

System	Catalyst type	Main product	Limiting potential (V)[a]	Level of theory	Publication year
Co@MoS_2	SAC	CH_4	-0.24 (*CO_2)	PBE-D3	2022 [9]
Cr-graphyne	SAC	CH_4	-0.29 (*CO_2)	PBE-vdWTS	2021 [10]
Mo-based 2D MOF	SAC	CH_4	-0.42 (*CO_2)	PBE-D2	2020 [11]
Co_2@N-doped graphene	DAC	C_2H_5OH	-0.45 (CO_2)	PBE-D3	2020 [12]
Cu_2@C_2N	DAC	CH_4	-0.23 (CO_2)	PBE-D2	2018 [13]
CuMn@N_6-C	DAC	CO	-0.43 (CO_2)	PBE+U	2020 [14]
NiSn@N-doped carbon nanosheet	DAC	HCOOH	-0.35 (CO_2)	PBE-D3	2021 [15]
Sn(001)	Pure metal	HCOOH	-0.50 (CO_2)	BEEF-vdW	2016 [16]
N-doped graphene	Metal-free catalyst	HCOOH	-0.44 (CO_2)	PBE-D2	2016 [17]
Bi-BTC-D	MOF	HCOOH	-0.22 (CO_2)	DFT-D with PBE	2022 [18]
Sn atoms supported on SnS_2 monolayer (this work)	SAC	HCOOH	-0.29 (CO_2)	HSE-D3 energetics over PBE-D3 geometry	

[a] The initial states used for evaluating the first electrochemical step in the studies are shown in parenthesis. *CO_2 indicates that the electrochemical step starts from an adsorbed CO_2 on the catalyst surface, and CO_2 indicates that the electrochemical step starts directly from a gas-phase CO_2.

Table S8. Adsorption free energy of reaction intermediates

	ΔG_{*OCHO} (eV)	ΔG_{*COOH} (eV)	ΔG_{*CO} (eV)	ΔG_{*H} (eV)
PBE-D3				
Ag-SAC	1.26	1.57	0.47	1.01
Cu-SAC	0.98	1.37	0.10	0.96
Sn-SAC	0.62	1.73	1.29	1.31
Zn-SAC	0.20	0.86	0.68	0.07
Ag-DAC	0.14	0.73	0.56	-0.06
Cu-DAC	-0.24	0.46	0.15	0.06
Sn-DAC	0.50	1.28	1.19	0.38
Zn-DAC	-0.76	0.10	0.86	0.16
HSE-D3				
Ag-SAC	1.01	1.33	0.05	0.87
Cu-SAC	0.76	1.16	-0.33	0.86
Sn-SAC	0.29	1.52	1.03	1.54
Zn-SAC	-0.12	0.53	0.29	-0.13
Ag-DAC	-0.22	0.29	0.18	-0.31
Cu-DAC	-0.58	0.07	-0.25	-0.12
Sn-DAC	0.21	-0.22	0.88	0.32
Zn-DAC	-1.03	-0.18	0.58	0.23

The ΔG_{ads} of each absorbate are generally higher at PBE-D3 level compared to HSE-D3. The average differences of ΔG_{ads} between the two theoretical levels are 0.297 eV for *OCHO, 0.449 eV for *COOH, 0.359 eV for *CO and 0.093 eV for *H. The trends in free energy changes are also reproduced between levels of PBE-D3 and HSE-D3.

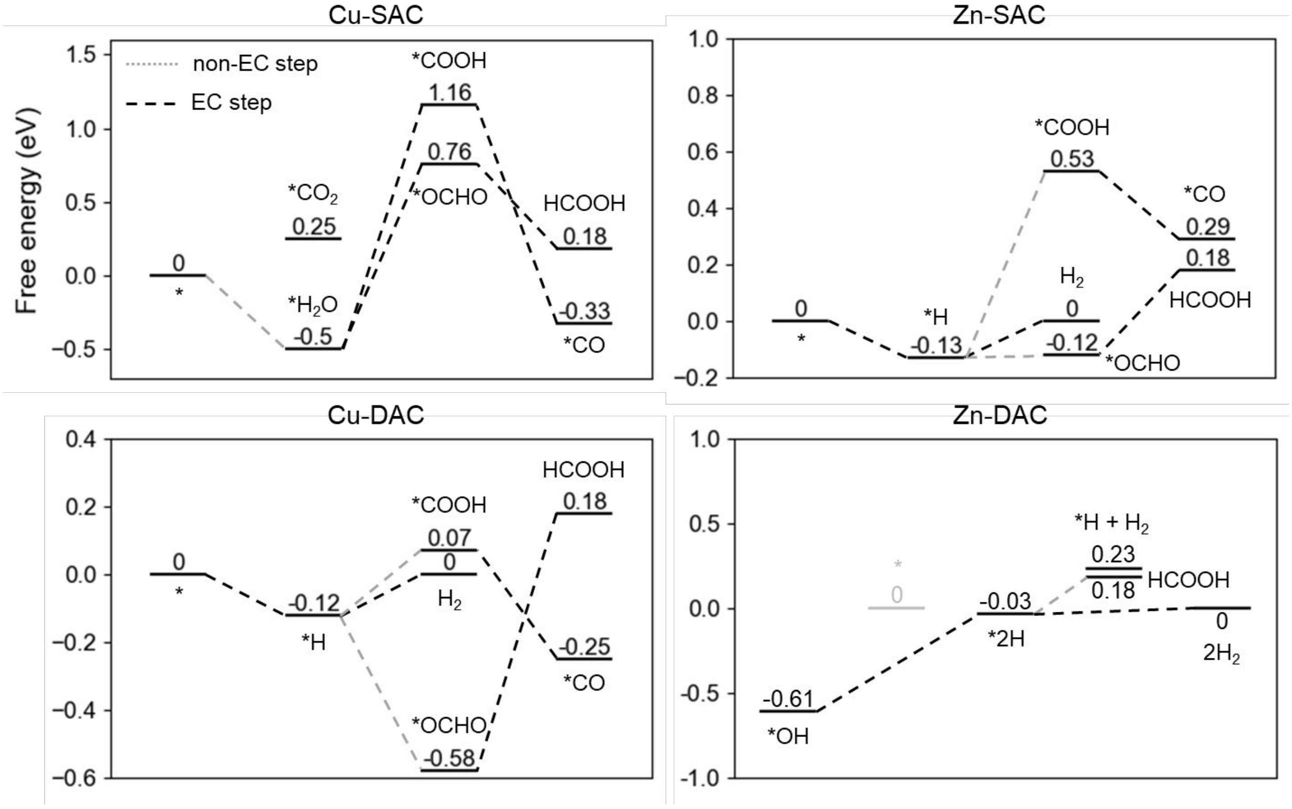

Figure S3. Free energy profiles of potentially favorable reaction pathways towards the formation of formic acid, *CO and H_2 on SACs and DACs with Cu and Zn. EC stands for "electrochemical" in the legend.

Table S9. Limiting potentials for HER on each catalyst

	SAC (V)	DAC (V)
Ag	-0.87	-0.31
Cu	-0.86	-0.12
Sn	-1.54	-0.32
Zn	-0.13	-0.23

3. Catalyst stability

Table S10. Formation energy (eV per atom) of SACs and DACs at different supercell sizes

Adsorption model:

Supercell size (n×n)	Ag		Cu		Sn		Zn	
	SAC	DAC	SAC	DAC	SAC	DAC	SAC	DAC
2	0.476	0.535	0.423	0.400	0.183	0.097	0.194	-0.420
3	0.515	0.504	0.548	0.472	0.364	0.382	0.265	0.037
4	0.487	0.520	0.513	0.514	0.319	0.433	0.267	0.215
5	0.489	0.512	0.519	0.510	0.305	0.397	0.229	0.192
6	0.487	0.516	0.520	0.509	0.296	0.386	0.224	0.179
7	0.489	0.509	0.524	0.506	0.286	0.376	0.226	0.177
8	0.487	0.506	0.521	0.503	0.284	0.372	0.214	0.167
9	0.489	0.506	0.525	0.502	0.283	0.369	0.215	0.166
10		0.505		0.501		0.369		0.164

Substitution model:

Supercell size (n×n)	Zn	
	SAC	DAC
3	1.700	0.301
4	1.718	0.314
5	1.740	0.296
6	1.740	Distortion
7	1.738	
8	1.735	
9	1.736	

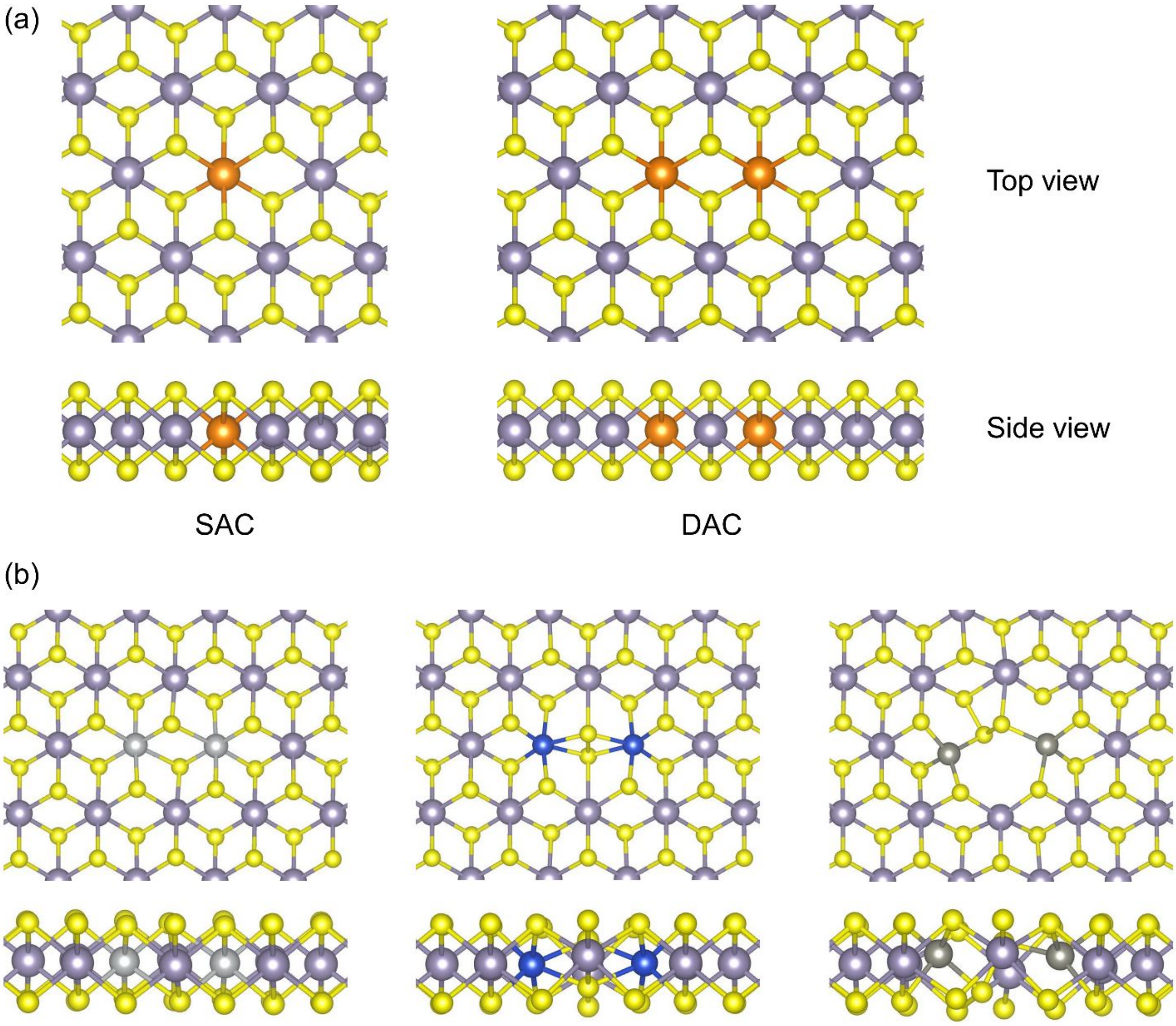

Figure S4. (a) Top and side views of substitution models on SnS_2 monolayer. (b) Optimised structures of substitutional model for DACs. Color of spheres represent: yellow, S; violet, Sn; orange, decorated metal atom; light grey, Ag; blue, Cu; dark grey, Zn.

Table S11. Formation energy per atom for substitution models

Metal	SAC (eV)	DAC (eV)
Ag	2.15	2.11
Cu	2.00	1.48
Zn	1.74	0.296

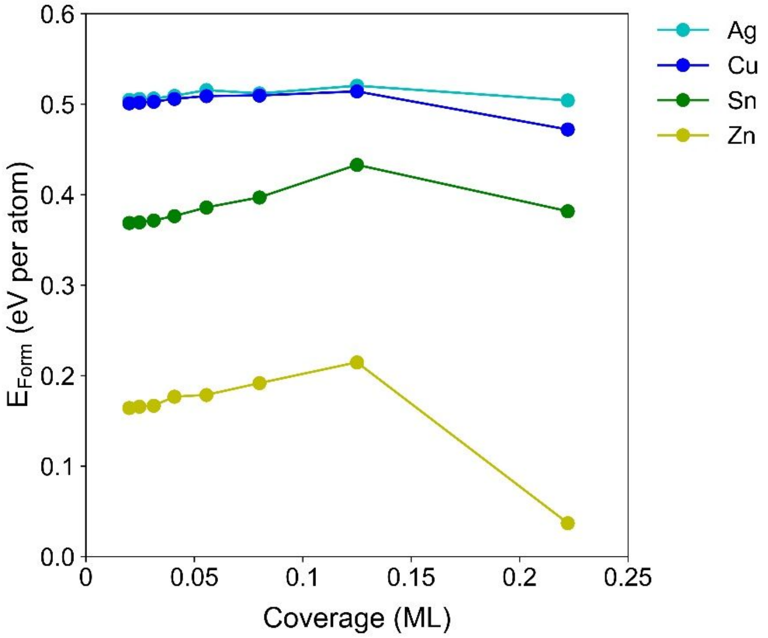

Figure S5. Trends in formation energy of DACs (adsorption models) vs. coverage.

4. Charges and electronic structures

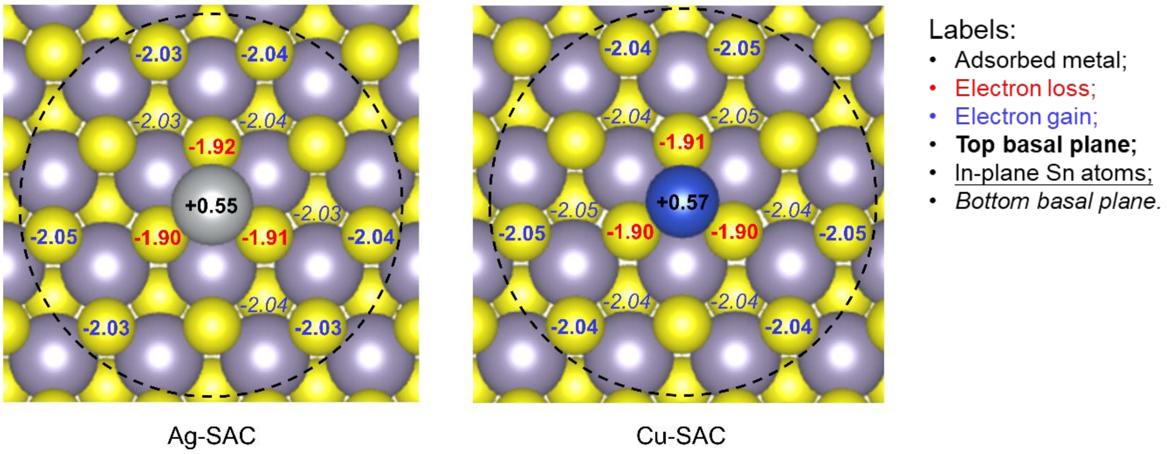

Figure S6. Atomic charges on Ag-SAC and Cu-SAC. Only atoms with charge variation above 0.03 |e| compared to pristine substrate are labelled. Dashed circle shows the charge redistribution area generated by one SAC metal center.

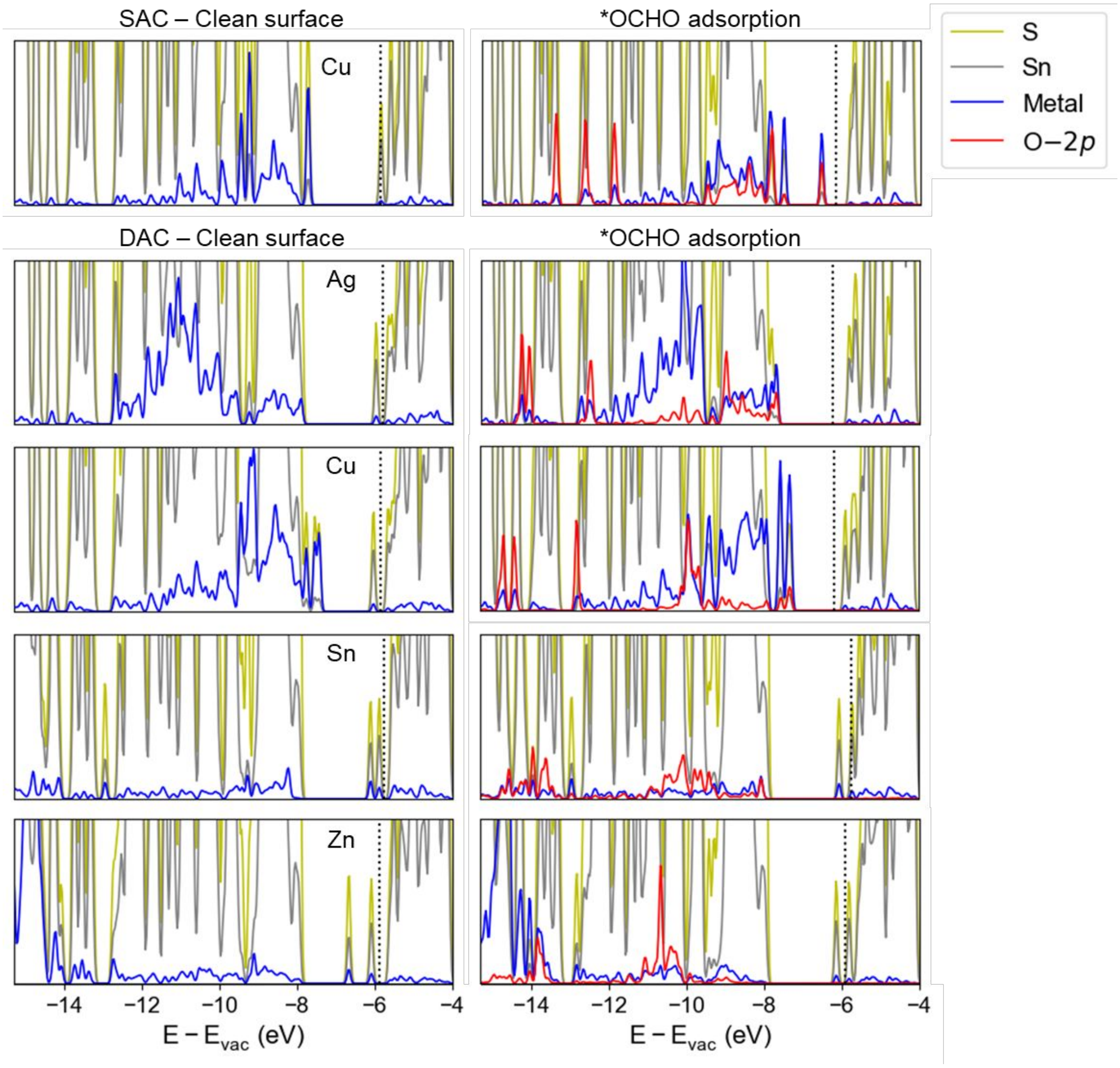

Figure S7. PDOS of clean surfaces and surfaces adsorbing *OCHO of Cu-SAC and all DACs.

5. References

(1) Guidon, M.; Hutter, J.; VandeVondele, J. Robust Periodic Hartree−Fock Exchange for Large-Scale Simulations Using Gaussian Basis Sets. *J Chem Theory Comput* **2009**, *5* (11), 3010–3021. https://doi.org/10.1021/ct900494g.

(2) Guidon, M.; Hutter, J.; VandeVondele, J. Auxiliary Density Matrix Methods for Hartree−Fock Exchange Calculations. *J Chem Theory Comput* **2010**, *6* (8), 2348–2364. https://doi.org/10.1021/ct1002225.

(3) Burton, L. A.; Whittles, T. J.; Hesp, D.; Linhart, W. M.; Skelton, J. M.; Hou, B.; Webster, R. F.; O'Dowd, G.; Reece, C.; Cherns, D.; Fermin, D. J.; Veal, T. D.; Dhanak, V. R.; Walsh, A. Electronic and Optical Properties of Single Crystal SnS_2: An Earth-Abundant Disulfide Photocatalyst. *J Mater Chem A Mater* **2016**, *4* (4), 1312–1318. https://doi.org/10.1039/C5TA08214E.

(4) Ye, G.; Gong, Y.; Lei, S.; He, Y.; Li, B.; Zhang, X.; Jin, Z.; Dong, L.; Lou, J.; Vajtai, R.; Zhou, W.; Ajayan, P. M. Synthesis of Large-Scale Atomic-Layer SnS_2 through Chemical Vapor Deposition. *Nano Res* **2017**, *10* (7), 2386–2394. https://doi.org/10.1007/s12274-017-1436-3.

(5) Kumagai, Y.; Burton, L. A.; Walsh, A.; Oba, F. Electronic Structure and Defect Physics of Tin Sulfides: SnS, Sn_2S_3, and SnS_2. *Phys Rev Appl* **2016**, *6* (1), 014009. https://doi.org/10.1103/PhysRevApplied.6.014009.

(6) Ullah, H.; Noor-A-Alam, M.; Shin, Y. Vacancy- and Doping-dependent Electronic and Magnetic Properties of Monolayer SnS_2. *Journal of the American Ceramic Society* **2020**, *103* (1), 391–402. https://doi.org/10.1111/jace.16739.

(7) Moret, S.; Dyson, P. J.; Laurenczy, G. Direct Synthesis of Formic Acid from Carbon Dioxide by Hydrogenation in Acidic Media. *Nat Commun* **2014**, *5* (1), 4017. https://doi.org/10.1038/ncomms5017.

(8) Peterson, A. A.; Abild-Pedersen, F.; Studt, F.; Rossmeisl, J.; Nørskov, J. K. How Copper Catalyzes the Electroreduction of Carbon Dioxide into Hydrocarbon Fuels. *Energy Environ Sci* **2010**, *3* (9), 1311. https://doi.org/10.1039/c0ee00071j.

(9) Ren, Y.; Sun, X.; Qi, K.; Zhao, Z. Single Atom Supported on MoS_2 as Efficient Electrocatalysts for the CO_2 Reduction Reaction: A DFT Study. *Appl Surf Sci* **2022**, *602*, 154211. https://doi.org/10.1016/j.apsusc.2022.154211.

(10) Fu, L.; Wang, R.; Zhao, C.; Huo, J.; He, C.; Kim, K.-H.; Zhang, W. Construction of Cr-Embedded Graphyne Electrocatalyst for Highly Selective Reduction of CO_2 to CH_4: A DFT Study. *Chemical Engineering Journal* **2021**, *414*, 128857. https://doi.org/10.1016/j.cej.2021.128857.

(11) Cui, Q.; Qin, G.; Wang, W.; Geethalakshmi, K. R.; Du, A.; Sun, Q. Novel Two-Dimensional MOF as a Promising Single-Atom Electrocatalyst for CO_2 Reduction: A Theoretical Study. *Appl Surf Sci* **2020**, *500*, 143993. https://doi.org/10.1016/j.apsusc.2019.143993.

(12) Chen, D.; Chen, Z.; Lu, Z.; Tang, J.; Zhang, X.; Singh, C. V. Computational Screening of Homo and Hetero Transition Metal Dimer Catalysts for Reduction of CO_2 to C_2 Products with High Activity and Low Limiting Potential. *J Mater Chem A Mater* **2020**, *8* (40), 21241–21254. https://doi.org/10.1039/D0TA05212D.

(13) Zhao, J.; Zhao, J.; Li, F.; Chen, Z. Copper Dimer Supported on a C_2N Layer as an Efficient Electrocatalyst for CO_2 Reduction Reaction: A Computational Study. *The Journal of Physical Chemistry C* **2018**, *122* (34), 19712–19721. https://doi.org/10.1021/acs.jpcc.8b06494.

(14) Luo, G.; Jing, Y.; Li, Y. Rational Design of Dual-Metal-Site Catalysts for Electroreduction of Carbon Dioxide. *J Mater Chem A Mater* **2020**, *8* (31), 15809–15815. https://doi.org/10.1039/D0TA00033G.

(15) Xie, W.; Li, H.; Cui, G.; Li, J.; Song, Y.; Li, S.; Zhang, X.; Lee, J. Y.; Shao, M.; Wei, M. NiSn Atomic Pair on an Integrated Electrode for Synergistic Electrocatalytic CO_2 Reduction. Angewandte Chemie International Edition **2021**, 60 (13), 7382–7388. https://doi.org/10.1002/anie.202014655.

(16) Yoo, J. S.; Christensen, R.; Vegge, T.; Nørskov, J. K.; Studt, F. Theoretical Insight into the Trends That Guide the Electrochemical Reduction of Carbon Dioxide to Formic Acid. ChemSusChem **2016**, 9 (4), 358–363. https://doi.org/10.1002/cssc.201501197.

(17) Liu, Y.; Zhao, J.; Cai, Q. Pyrrolic-Nitrogen Doped Graphene: A Metal-Free Electrocatalyst with High Efficiency and Selectivity for the Reduction of Carbon Dioxide to Formic Acid: A Computational Study. Physical Chemistry Chemical Physics **2016**, 18 (7), 5491–5498. https://doi.org/10.1039/C5CP07458D.

(18) Zhang, X.; Zhang, Y.; Li, Q.; Zhou, X.; Li, Q.; Yi, J.; Liu, Y.; Zhang, J. Highly Efficient and Durable Aqueous Electrocatalytic Reduction of CO_2 to HCOOH with a Novel Bismuth–MOF: Experimental and DFT Studies. J Mater Chem A Mater **2020**, 8 (19), 9776–9787. https://doi.org/10.1039/D0TA00384K.
